# Synthesis, Molecular Docking and Antiplasmodial Activities of New Tetrahydro-β-Carbolines

**DOI:** 10.3390/ijms222413569

**Published:** 2021-12-17

**Authors:** Anna Jaromin, Beata Gryzło, Marek Jamrozik, Silvia Parapini, Nicoletta Basilico, Marek Cegła, Donatella Taramelli, Agnieszka Zagórska

**Affiliations:** 1 Department of Lipids and Liposomes, Faculty of Biotechnology, University of Wrocław, Joliot-Curie 14a, 50-383 Wroclaw, Poland; 2 Faculty of Pharmacy, Jagiellonian University Medical College, 30-688 Krakow, Poland; beata.gryzlo@uj.edu.pl (B.G.); marek.jamrozik@doctoral.uj.edu.pl (M.J.); marek.cegla@uj.edu.pl (M.C.); 3 Dipartimento di Scienze Biomediche per la Salute, Università degli Studi di Milano, 20133 Milan, Italy; silvia.parapini@unimi.it; 4 Dipartimento di Scienze Biomediche, Chirurgiche e Odontoiatriche, Università degli Studi di Milano, 20133 Milan, Italy; nicoletta.basilico@unimi.it; 5 Dipartimento di Scienze Farmacologiche e Biomolecolari, Università degli Studi di Milano, 20133 Milan, Italy; donatella.taramelli@unimi.it

**Keywords:** tetrahydro-β-carbolines, *Plasmodium falciparum* (*P. falciparum*), antimalarial, antiparasitic agents, cytotoxicity, hemolysis, molecular docking

## Abstract

Malaria is still one of the most dangerous infectious diseases and the emergence of drug resistant parasites only worsens the situation. A series of new tetrahydro-β-carbolines were designed, synthesized by the Pictet–Spengler reaction, and characterized. Further, the compounds were screened for their in vitro antiplasmodial activity against chloroquine-sensitive (D10) and chloroquine-resistant (W2) strains of *Plasmodium falciparum*. Moreover, molecular modeling studies were performed to assess the potential action of the designed molecules and toxicity assays were conducted on the human microvascular endothelial (HMEC-1) cell line and human red blood cells. Our studies identified *N*-(3,3-dimethylbutyl)-1-octyl-2,3,4,9-tetrahydro-1H-pyrido[3,4-b] indole-3-carboxamide (**7**) (a mixture of diastereomers) as the most promising compound endowed with the highest antiplasmodial activity, highest selectivity, and lack of cytotoxicity. In silico simulations carried out for (1*S*,3*R*)-**7** provided useful insights into its possible interactions with enzymes essential for parasite metabolism. Further studies are underway to develop the optimal nanosized lipid-based delivery system for this compound and to determine its precise mechanism of action.

## 1. Introduction

Malaria is an infectious disease caused by the protozoan parasite of the genus *Plasmodium*, with *Plasmodium falciparum* and *Plasmodium vivax* being predominantly responsible for mortality and morbidity. In 2019, the global tally of malaria cases was 229 million, an annual estimate that has remained virtually unchanged over the last 4 years [1]. Despite the COVID-19 pandemic, an analysis of malaria prevention showed that the 2020 campaigns to control and eradicate malaria were realized as planned. Lastly, in October of 2021, the WHO has recommended Mosquirix, the RTS,S/AS01 malaria vaccine, for the prevention of *P. falciparum* malaria in children living in regions with moderate to high transmission [2]. However, disruptions to continued access to effective antimalarial treatment could lead to considerable loss of life [3].

First-line malaria treatments include several artemisinin-based combination therapies (ACTs), such as artemether-lumefantrine (AL), artesunate-amodiaquine (AS-AQ), artesunate-sulfadoxine-pyrimethamine (AS+SP), artesunate-mefloquine (AS-MQ), and dihydroartemisinin-piperaquine (DHA-PPQ). The malaria problem is evolving, dynamic, and diverse, mainly due to the intrinsic ability of *P. falciparum* to acquire resistance against drugs. *P. falciparum* has developed resistance to nearly all currently available antimalarial drugs, such as sulfadoxine/pyrimethamine, mefloquine, halofantrine, and quinine [4]. The observed decrease in the effectiveness of artemisinin is correlated with mutation in the *P. falciparum* Kelch13 gene [5]. In addition, there is resistance against artemisinin partner drugs, such as mefloquine and piperaquine [6,7]. Thus, there is an urgent need to find a replacement for artemisinin or novel artemisinin partner-drugs active against known-resistant strains. Currently, all new molecules are tested against a wide variety of resistant laboratory strains of *P. falciparum*, and activity in these assays is a key requirement for further evaluation.

**Figure 1:**
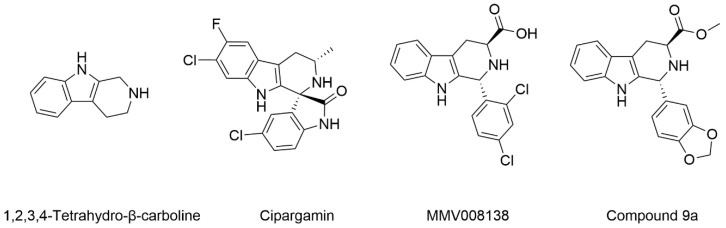
Examples of THβC derivatives with potent antimalarial activity.

The 1,2,3,4-tetrahydro-β-carboline (9H-1,2,3,4-tetrahydropyrido(3,4-b)indole, THβC) core (Figure 1) is a privileged structure found in many antimalarial drug candidates and represents an important scaffold for the discovery of novel potent antimalarials Among the THβC, C1−C3 substituted derivatives were reported as having potent antimalarial activity [8]. Cipargamin (KAE609, NITD609) (Figure 1) is a novel spiroindolone-class drug for the treatment of malaria. The compound displayed low nanomolar 50% inhibitory concentration (IC_50_) values (range 0.5–1.4 nM), with no evidence of diminished potency against drug-resistant strains. Cipargamin acts on the P-type Na^+^ ATPase (*Pf*ATP4) of *P. falciparum*, disrupting its Na^+^ homeostasis. This mechanism is distinct from that of existing antimalarial drugs. Currently, cipargamin is undergoing a phase 2 clinical trial [9].

Similarly, compound MMV008138 (Figure 1) exhibits potent antimalarial activity and inhibits the growth of the *P. falciparum* Dd2 strain with an IC_50_ of 250 nM [10]. Gorki et al. explored the antimalarial activity of a β-carboline derivative, compound **9a** (Figure 1), against *P. falciparum*. Compound **9a** inhibited both the 3D7 and RKL-9 strains of *P. falciparum* with an IC_50_ < 2.86 μM, respectively, was nontoxic to normal dermal fibroblasts, and its selectivity index was >10 against both strains [11]. Further, Gellis et al. reported on a series of 1-phenyl-substituted-β-carboline derivatives with significant antimalarial activity (0.7 < IC_50_ < 1.7 mM) against the W2 multidrug-resistant strain of *P. falciparum* [12].

**Figure 2:**
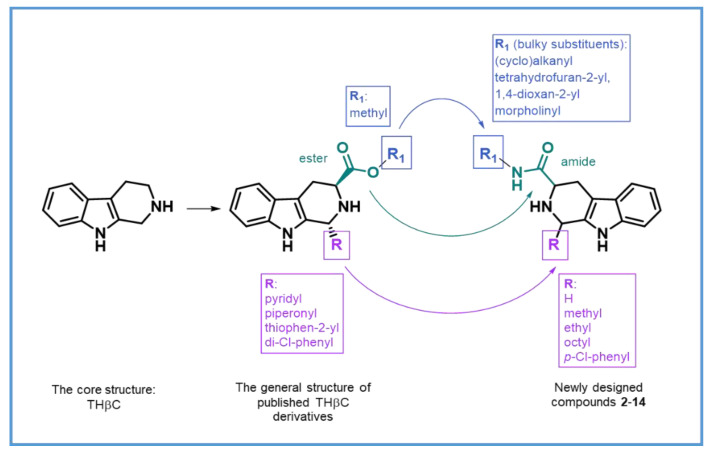
Modification of THβC and general structure of the new compounds (**2**–**14**).

Considering these promising antimalarial activities of various THβC derivatives, we designed and synthesized a new series of compounds with aromatic and/or aliphatic side chains in the 1-position and an amide bond in the 3-position of THβC (Figure 2). The known THβC derivatives active against the drug-resistant *P. falciparum* contain mostly bulky aromatic or heteroaromatic substituents (such as piridyl, piperonyl, thiophene, or di-chlorophenyl moieties) at the C1 position and small methyl esters at the C3 position. Consequently, we decided to change the physicochemical properties of the substituents at the C1 and C3 positions, namely, from bulky to small at the C1 position and from small to bulky at the C3 position. The target compounds in the further biological investigations that were conducted, were used as undetermined mixtures of enantiomers and as pure diastereomers or as a mixture of diastereomers.

The synthesized compounds were evaluated for in vitro activity against D10 (CQ-sensitive) and W2 (CQ-resistant) strains of *P. falciparum*. Subsequently, the cytotoxic activity on human microvascular endothelial cells and hemolytic effects on human erythrocytes were also investigated, to obtain more information concerning their safety against mammalian cells. Moreover, compounds **2**–**14** were also characterized by their physicochemical parameters (Log D). Next, we focused on the probable mechanism of action of the most active compound. Thus, molecular modeling studies were performed to assess the potential binding modes of one enantiomer of compound **7** (that is (1*S*,3*R*)-**7**) with a series of different enzymes essential for parasite metabolism. Phosphoethanolamine methyltransferase (PMT) plays a critical function in parasite development and differentiation but is absent in mammals. PMT catalyzes the synthesis of phosphatidylcholine—the major phospholipid constituent of the membranes of parasites during the sexual and asexual stages of *Plasmodium* [13]. Lactate dehydrogenase (LDH) is synthesized by parasites in the blood-stage of malaria as the terminal enzyme in the glycolytic pathway of *Plasmodium* [14]. Cytosolic malate dehydrogenase (MDH) converts malate to oxaloacetate and, as a result, generates NADH or NADPH, two reducing equivalents to the respiratory chain of *Plasmodium* [15]. Falcipain-2 (FP2) and falcipain-3 (FP3) are critical hemoglobinases of *P. falciparum*, which catalyze the degradation of hemoglobin into hemozoin [16].

For this, five different enzymes of *P. falciparum* were selected for docking studies with the view of investigating a possible multitargeting mode of action. Such a strategy would constitute a promising solution to *P. falciparum* drug resistance. To our knowledge, this is the first study describing the syntheses of this type of tetrahydro-β-carboline series as well as their antiplasmodial activities and the in silico approach used to study their probable mechanisms of action on this parasite.

## 2. Results and Discussion

### 2.1. Chemistry

**Figure 3:**
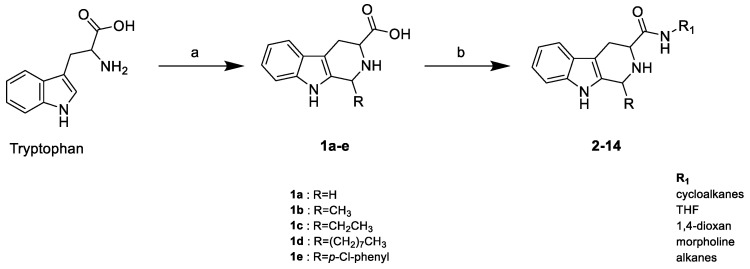
Synthesis of compounds **2**–**14**. Reagents and conditions: (**a**) RCHO, H_2_SO_4_, H_2_O, reflux or rt, 20 h; (**b**) amine, DMAP, EDC, HOBT, pyridine, DCM, reflux, 24 h.

The synthesis of the designed THβC derivatives (**2**–**14**) commenced with the Pictet–Spengler reaction of racemic tryptophan and an appropriate aldehyde in the presence of sulfuric acid (H_2_SO_4_) in water (Figure 3) [17]. The resultant 1-substituted-tetrahydro-β-carboline-3-carboxylic acids (**1a**–**e**) were further reacted with diverse amines in the presence of 4-dimethylaminopyridine (DMAP), 1-ethyl-3-(3-dimethylaminopropyl) carbodiimide (EDC), 1-hydroxybenzotriazole (HOBt), and pyridine. The final compounds were purified by crystallization from hexane or by column chromatography. The structures and purity of the newly synthesized compounds were characterized by ^1^H NMR, ^13^C NMR spectroscopy, and UPLC/MS spectrometry. Compounds were isolated as an undetermined mixture of enantiomers (**3RS-2**, **1RS**,**3SR** for **8**–**11** and **14**), pure diastereomers (**1RS**,**3RS-13**, **1RS**,**3SR-13**) or as a mixture of diastereomers (**1**, **3**–**7**, **12**) with various diastereoselectivity (ds) (Section 3 and Section 3.1).

The applied synthetic methods facilitated the synthesis of intermediates (**1a**–**e**), and compounds (**2**–**14**), with yields of 15–74% and the purity of the final compounds above 95%.

### 2.2. Log D Calculation

Compounds **2**–**14** are partially ionized; thus, the distribution coefficients D (Log D) were calculated at fixed pH. The chosen pH (7.4, 7.2, and 5.5) represent the physiological conditions found in the blood, human erythrocyte cytoplasm, and *P. falciparum* food vacuoles. The data in Table 1 show that, for Log D at pH 7.4, the compounds fall in the range 1.73–5.65, and in the range of 1.59–5.53 at pH 7.2, whereas at pH 5.5, the compounds were in the range of 0.05–3.93. Only compound **3** displayed a gradient of Log D similar to CQ, however, the values of Log D for compound **3** were almost twice as large as those for CQ itself.

### 2.3. Antiplasmodial Activities

Next, the screening of antiplasmodial activities in in vitro parasite cultures was performed using standard procedures. For this purpose, we tested the susceptibility of two strains of *P. falciparum* differing in resistance to CQ, namely D10 (chloroquine-sensitive) and W2 (chloroquine-resistant). The inhibitory effect on the growth of these strains, expressed as IC_50_ values, is presented in Table 2. The synthesized derivatives have IC_50_ values in the range of 4.00 ± 0.53–35.36 ± 4.86 µM. The highest antiplasmodial activity among the tested series was shown by compound **7**, which is also characterized by one of the highest Log D values in the series of compounds. Moreover, many compounds have a similar magnitude of inhibitory activity against the two *P. falciparum* strains, as reported for other tetrahydro-β-carboline derivatives by Eagon et al. [18]. The important fact is also that, except for compound **6**, the determined values of IC_50_ are lower for the CQ-resistant strain in comparison to the CQ-sensitive strain, reflected in their RI (resistance index) values. This could suggest superior sensitivity of the designed compounds towards the CQ-resistant parasite strain. The obtained results are very encouraging, especially since malaria control is threatened by the emergence of drug resistance to the artemisinin derivatives [19]. The data also indicate a different relationship between chemical structure and antiplasmodial activity that is not directly related to Log D values and the types of chemical substituents.

### 2.4. Biocompatibility Studies

Results from the preliminary antiplasmodial activity screening highlighted the necessity for the evaluation of the safety of these agents to human cells. Hence, we chose two model cell systems: the human microvascular endothelial (HMEC-1) cell line and human erythrocytes, to obtain greater insights into their cell toxicity potential. In these studies, we focused only on derivatives that had exhibited the highest antiplasmodial activities, namely **3**, **6**, **7**, **8**, **9**, **12**, **1RS**,**3RS-13**, and **1RS**,**3SR-13**. The results concerning their cytotoxic effects on HMEC-1 cells as well as their calculated selectivity index (SI), namely the ratio between the IC_50_ values on the endothelial cells and that on the *Plasmodium* strains, are summarized in Table 3. Based on the analysis of the obtained results, it can be concluded that the determined IC_50_ values fall within a fairly wide range from 17.95 ± 9.46 to 157.18 ± 42.50 µM, even though they all have their IC_50_ in the range of low micromolar concentrations against both strains of *Plasmodium*. A similar phenomenon has already been described for methoxy-thiazinoquinones by Imperatore et al. [20]. Interestingly, the most active compound against the parasite (**7**) is also safe to HMEC-1 cells and is highly selective, also exhibiting the highest SI value of the whole compound series.

We also sought to investigate the hemolytic potential of the synthetized compounds. For this experiment, we selected three compounds, namely **3**, **7**, and **12**, which exhibited the highest selectivity. We incubated these compounds at a concentration of 10 µM, with human erythrocytes, and then, by measuring the released hemoglobin, we assessed their potential harmful effects on these cells. The measured level of hemolysis was less than 5% for all agents, which proves that the compounds are not hemolytic. It is worth emphasizing at this point that this type of test is extremely important to confirm that the observed inhibitory activity against *Plasmodium* is a result of a compound acting directly upon it and not as a result of red blood cell lysis. Taking into account the fact that the tested concentration of **7** in this test was, respectively, 2.2 and 2.5 times higher than the IC_50_ determined for the *Plasmodium* strains, we can conclude that the observed effect on the parasite is not due to activity on erythrocytes. In conclusion, compound **7** is, therefore, biocompatible, since no hemolytic effects were detected in the mammalian cells tested.

### 2.5. Molecular Modeling

Molecular modeling studies were performed to evaluate the possible mechanism of action of the one enantiomer of compound **7** namely (1*S*,3*R*)-3-[(3,3-dimethylbutyl)carbamoyl]-1-octyl-1*H*,2*H*,3*H*,4*H*,9*H*-pyrido[3,4-*b*]indol-2-ium (*trans*); (compound (1*S*,3*R*)-**7**). The LigPrep tool and MarvinSketch software indicated that, at pH 7.4, 73% of compound (1*S*,3*R*)-**7** molecules exist in the form where the 1,2,3,6-tetrahydropyridine ring has a protonated amine group, whereas 27% of molecules remain uncharged. Faced with such a clear disproportion, we assumed that the protonated form is responsible for most of the physiological effect and, consequently, only this form was used in the docking simulations. Compound (1*S*,3*R*)-**7** was docked to the following series of enzymes essential for the functioning of *P. falciparum:* phosphoethanolamine methyltransferase (PMT), falcipain-2 (FP2), falcipain-3 (FP3), lactate dehydrogenase (LDH), and malate dehydrogenase (MDH). Moreover, compound (1*S*,3*R*)-**7** formed molecular interactions with the amino acid residues of these enzymes, as observed in the originally co-crystallized ligands.

**Figure 4:**
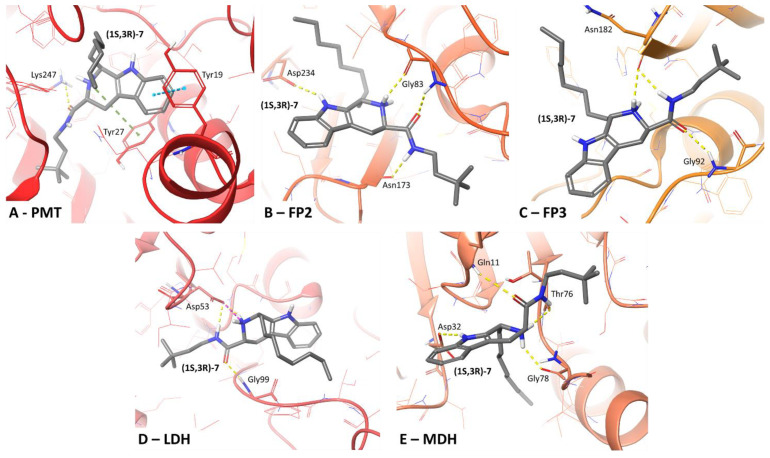
Molecular interactions formed between compound (1*S*,3*R*)-**7** and selected molecular targets phosphoethanolamine methyltransferase (PMT) (**A**); falcipain-2 (FP2) (**B**); falcipain-3 (FP3) (**C**); lactate dehydrogenase (LDH) (**D**); and malate dehydrogenase (MDH) (**E**). Amino acid residues within 4 Å from the ligand are displayed as thin sticks; amino acid residues engaged in ligand binding by ionic bond (dotted pink lines), H-bond (dotted yellow lines), π-cation interaction (dotted green lines), and π- π interaction (dotted blue lines) are displayed as bold sticks.

In the complex of compound (1*S*,3*R*)-**7** and PMT that was obtained by IFD simulations (Figure 4A), a π-cation interaction between Tyr27 of PMT and the protonated amine group of tetrahydropyridine, as well as hydrogen-bonding between the side chain of Lys247 of PMT and the oxygen in the amide moiety of compound (1*S*,3*R*)-**7** were observed. Moreover, both mentioned amino acid residues participated in forming interactions with phosphocholine in the PMT crystal. In addition, a π–π interaction was observed between Tyr19 of PMT and the aromatic benzene ring from the β-carboline moiety. This additional interaction was not observed in the PMT crystal structure.

The obtained complex of compound (1*S*,3*R*)-**7** and falcipain-2 (Figure 4B) formed H-bonds between the main chain of Gly83 in PMT and the protonated amine group of tetrahydropyridine and the oxygen in the amide moiety of compound (1*S*,3*R*)-**7**. A hydrogen-bond was also observed between the main chain of Asn173 of PMT and the hydrogen on the amide grouping in compound (1*S*,3*R*)-**7**. All the amino acid residues discussed previously formed interactions with epoxysuccinate in the FP2 crystal. However, the H-bond between the side chain of Asp234 and the amine group of the center ring of β-carboline was not observed in the FP3 crystal structure.

The complex of FP3 (Figure 4C) and compound (1*S*,3*R*)-**7** formed H-bonds between the main chain of Asn182 and the protonated amine group of tetrahydropyridine, as well as with hydrogen from the amide of compound (1*S*,3*R*)-**7**. In addition, a hydrogen bond between the main chain of Gly92 of FP3 and the amide’s oxygen of compound (1*S*,3*R*)-**7** was observed. It is noteworthy that all the mentioned amino acid residues participated in interactions with leupeptin in the FP3 crystal.

An ionic bond (salt bridge) was observed between the side chain of Asp53 in LDH and the protonated amine group of tetrahydropyridine of compound (1*S*,3*R*)-**7**, as well as a hydrogen bond between the side chain of Asp53 and the hydrogen of the amide group of compounds (1*S*,3*R*)-**7**, and an additional H-bond identified between the main chain of Gly99 and the oxygen of the amide group of compounds (1*S*,3*R*)-**7** (Figure 4D). All the mentioned amino acid residues participated in forming interactions with NADH in the LDH crystal.

Compound (1*S*,3*R*)-**7** (Figure 4E) formed a hydrogen bond between the main chain of MDH’s Gly78 and the protonated amine group of tetrahydropyridine, along with an H-bond between the main chain of Thr76 and the protonated amine group of tetrahydropyridine and the hydrogen of the amide moiety of compound (1*S*,3*R*)-**7**. A further H-bond exists between the main chain of Gln11 and the oxygen on the amide moiety of compound (1*S*,3*R*)-**7**, and an H-bond between the side chain of Asp32 and the amine group of the center ring of β-carboline of compound (1*S*,3*R*)-**7**. Among the mentioned amino-acid interactions, only Thr76 was not involved in forming interactions with NAD in the 6R8G crystal.

Results from IFD revealed that each H-bond donor/acceptor fragment present in compound (1*S*,3*R*)-**7**, such as the amine group of the center ring of β-carboline, the protonated amine group on tetrahydropyridine, and the oxygen, nitrogen, and hydrogen atoms of the amide moiety of compound (1*S*,3*R*)-**7**, were involved in forming an interaction with at least one amino-acid target in the group of enzymes investigated. Moreover, the aliphatic chains of compound (1*S*,3*R*)-**7** additionally stabilized the position of the entire molecule within the enzymes. Observations from in silico simulations highlight that compound (1*S*,3*R*)-**7** has structural features enabling it to interact with five described molecular targets within sites occupied originally by other ligands (products/inhibitors/cofactors). The results are a good starting point for further in vitro evaluation of compound (1*S*,3*R*)-**7**.

## 3. Materials and Methods

### 3.1. General Remarks

Commercially available reagents were purchased from Merck-Sigma-Aldrich (Poznań, Poland), Acros Organics (Thermo Fisher Scientific, Waltham, MA, USA), or ChemPur (Piekary Śląskie, Poland) and were used without further purification. Purification of chemical compounds by column chromatography was carried out using silica gel mesh: 0.063–0.200 μm (Sigma-Aldrich; Poznań, Poland) as a stationary phase. The reactions were monitored by thin-layer chromatography on aluminum sheets precoated with silica gel 60 F_254_ (Merck; Darmstadt, Germany). Compounds were visualized with UV light (254 nm). In addition, chromatograms were stained in a 0.5% solution of ninhydrin in n-propanol or a solution of 5% (NH_4_)_6_Mo_7_O_24_ and 0.2% Ce(SO_4_)_2_ in 5% H_2_SO_4_. The retardation factor R_*f*_ was defined using the following solvent systems: S_1_ (DCM/methanol/acetic acid, 8:2:0.5 *v*/*v*/*v*), S_2_ DCM/methanol (95:5, *v*/*v*), S_3_ DCM/EtOAc (9:1, *v*/*v*), S_4_ DCM/MeOH (97.5:2.5 *v*/*v*), S_5_ DCM/EtOAc (14:1 *v*/*v*). ^1^H NMR and ^13^C NMR spectra were recorded using an FT-NMR 500 MHz spectrometer (Joel Ltd., Akishima, Tokyo, Japan). The chemical shifts (δ) are reported in ppm and were calculated concerning the frequency of the deuterium field stabilization signal. The coupling constant *J* is reported in Hertz. Signal multiplets are represented by the following abbreviations: s (singlet), brs (broad singlet), d (doublet), dd (doublet of doublets), dt (doublet of triplets), t (triplet), q (quintet), m (multiplet). (Supplementary Table S1). Diastereoselectivity was characterized by ds (major/minor) according to ^1^H NMR spectra [22]. UPLC separations were carried out according to the procedures described elsewhere [23].

Medium and supplements for *P. falciparum* culture and cell culture (RPMI 1640 Medium, Glutamine, Hepes Buffer, Fetal calf serum) were from EuroClone (Milan, Italy). AlbuMax and MCDD 131 medium were from Invitrogen (Milan, Italy). Unless stated otherwise, all reagents were from Sigma Italia (Milan, Italy).

### 3.2. Synthetic Procedures

#### 3.2.1. General Method for Synthesis of the 1-Substituted-tetrahydro-β-Carboline-3-Carboxylic Acids (**1a**–**e**)

According to the literature, a mixture of 0.5 N H_2_SO_4_ (2.5 mL) and H_2_O (200 mL), d,l-tryptophan (2.04 g, 10 mmol), and appropriate aldehydes (30 mmol) was stirred at room temperature overnight and the synthesis products detected by TLC. The precipitate was filtered and washed well with H_2_O and dried in a vacuum. The material was used without further purification for the following steps.

#### 3.2.2. General Method for Synthesis of the 3-Carboxamide Derivatives of 1-Substituted-tetrahydro-β-carboline-3-carboxylic Acids (**2**–**14**)

The appropriate 1-substituted-tetrahydro-β-carboline-3-carboxylic acid (**1a–e**) (1 equiv) suspensions in dichloromethane (DCM) with EDC (1.3 equiv), DMAP (0.5 equiv), HOBT (1.3 equiv), pyridine (0.05 equiv), and a relevant amine (1 equiv) were refluxed for 18 h. When the reactions were completed, the mixtures were extracted with CHCl_3_/isopropanol (3:1 *v*/*v*; 3 × 10 mL). The combined organic fractions were dried over Na_2_SO_4_ and evaporated under vacuum. The crude products were purified by crystallization in hexane or by column chromatography.

- *N*-cyclooctyl-2,3,4,9-tetrahydro-1*H*-pyrido[3,4-*b*]indole-3-carboxamide (**2**): (**3RS**)

Compound **2** was prepared using 2,3,4,9-tetrahydro-1*H*-pyrido[3,4-*b*]indole-3-carboxylic acid (**1a**) (0.25 mmol, 0.058 g) and EDC (0.325 mmol, 0.062 g), HOBT (0.325 mmol, 0.043 g), DMAP (0.125 mmol, 0.015 g), pyridine (0.025 mmol, 2 µL) and cyclooctanamine (0.25 mmol, 38 μL) in 8 mL DCM. The product was purified by column chromatography over silica gel and obtained as a crystallized oil with a yield of 40% (R*_f_* = 0.29 (S_2_)). LC/MS: C_20_H_27_N_3_O (98%) *m*/*z*: 326.22, found: 326.07. ^1^H NMR (500 MHz, CDCl_3_) δ 8.05 (br. s., 1H), 7.49 (d, *J* = 8.0 Hz, 1H), 7.32–7.28 (m, 1H), 7.19–7.13 (m, 1H), 7.12–7.08 (m, 1H), 7.04 (d, *J* = 8.0 Hz, 1H), 4.07–3.98 (m, 3H), 3.52 (dd, *J* = 4.87 Hz, 10.60 Hz, 1H), 3.23 (dd, *J* = 4. 6 Hz, 15. 5 Hz, 1H), 2.82–2.74 (m, 1H), 1.92–1.81 (m, 2H), 1.69 (d, *J* = 8.6 Hz, 2H), 1.61–1.51 (m, 8H). ^13^C NMR (126 MHz, CDCl_3_) δ 171.5, 136.1, 132.3, 127.4, 121.8, 119.6, 118.1, 110.9, 108.6, 57.5, 49.1, 43.0, 32.3, 27.3, 25.5, 24.8, 23.8.

- (1-methyl-2,3,4,9-tetrahydro-1*H*-pyrido[3,4-*b*]indol-3-yl)(piperidin-1-yl)methanone (**3**): (**1RS**,**3SR**)-**3/**(**1RS**,**3RS**)-**3**

Compound **3** was prepared using 1-methyl-2,3,4,9-tetrahydro-1*H*-pyrido[3,4-b]indole-3-carboxylic acid (**1b**) (0.25 mmol, 0.058 g) and EDC (0.325 mmol, 0.062 g), HOBT (0.325 mmol, 0.043 g), DMAP (0.125 mmol, 0.015 g), pyridine (0.025 mmol, 2 µL) and piperidine (0.25 mmol, 26 μL) in 8 mL DCM. The product was purified by column chromatography over silica gel and obtained as a yellowish solid with a yield of 72% (R*_f_* = 0.25 (S_2_)). LC/MS: C_18_H_23_N_3_O (95%) *m*/*z*: 298.18, found: 298.28. ds: (**1RS**,**3SR**)-**3/**(**1RS**,**3RS**)-**3** = 70:3. ^1^H NMR (500 MHz, CDCl_3_) δ 8.62–8.54 (m, 1H), 7.42 (d, *J* = 7.5 Hz, 1H), 7.31 (s, 1H), 7.10 (d, *J* = 7.16 Hz, 1H), 7.08–7.03 (m, 1H), 4.30–4.23 (m, 1H), 4.08–4.03 (m, 1H), 3.59 (t, *J* = 5.3 Hz, 1H), 3.48–3.43 (m, 1H), 2.88–2.77 (m, 2H), 2.15 (s, 3H), 1.69–1.48 (m, 8H) *NH* proton was not detected. ^13^C NMR (126 MHz, CDCl_3_) δ 171.1, 136.5, 135.9, 126.9, 121.6, 119.4, 117.7, 112.1, 111.0, 107.0, 53.4, 46.5, 42.9, 30.9, 25.4, 24.4, 20.0.

- *N*-cyclohexyl-1-methyl-2,3,4,9-tetrahydro-1*H*-pyrido[3,4-*b*]indole-3-carboxamide (**4**): (**1RS**,**3SR**)-**4/**(**1RS**,**3RS**)-**4**

Compound **4** was prepared using 1-methyl-2,3,4,9-tetrahydro-1*H*-pyrido[3,4-*b*]indole-3-carboxylic acid (**1b**) (0.25 mmol, 0.058 g) and EDC (0.325 mmol, 0.062 g), HOBT (0.325 mmol, 0.043 g), DMAP (0.125 mmol, 0.015 g), pyridine (0.025 mmol, 2 µL) and cyclohexanamine (0.25 mmol, 28 μL) in 8 mL DCM. The product was purified by column chromatography over silica gel and obtained as a yellowish solid with a yield of 74% (R*_f_* = 0.11 (S_2_)). LC/MS: C_19_H_25_N_3_O (96%) *m*/*z*: 311.20, found 311.15. ds: (**1RS**,**3SR**)-**4/**(**1RS**,**3RS**)-**4** = 10:4. ^1^H NMR (500 MHz, CDCl_3_) δ 8.40 (s, 1H), 7.47 (d, *J* = 8.0 Hz, 1H), 7.31 (d, *J* = 8.0 Hz, 1H), 7.18–7.08 (m, 2H), 7.07–6.98 (m, 1H), 4.21–4.10 (m, 1H), 3.89–3.77 (m, 1H), 3.54 (dd, *J* = 4.6, 11.5 Hz, 1H), 3.28 (ddd, *J* = 2.0, 4.6, 15.8 Hz, 1H), 2.69 (ddd, *J* = 2.9 Hz, 11.3 Hz, 15.6 Hz, 1H), 2.01–1.90 (m, 2H), 1.78–1.69 (m, 2H), 1.63 (td, *J* = 3.6 Hz, 12.9 Hz, 1H), 1.49 (d, *J* = 6.9 Hz, 3H), 1.45–1.31 (m, 3H), 1.30–1.11 (m, 3H). ^13^C NMR (126 MHz, CDCl_3_) δ 172.1, 137.1, 136.2, 127.4, 121.8, 119.6, 118.3, 111.1, 108.4, 58.0, 49.5, 48.0, 33.2, 25.5, 20.4.

- *N*-(cyclohexylmethyl)-1-methyl-2,3,4,9-tetrahydro-1*H*-pyrido[3,4-*b*]indole-3-carboxamide (**5**): (**1RS**,**3SR**)-**5/**(**1RS**,**3RS**)-**5**

Compound **5** was prepared using 1-methyl-2,3,4,9-tetrahydro-1*H*-pyrido[3,4-*b*]indole-3-carboxylic acid (**1b**) (0.25 mmol, 0.058 g) and EDC (0.325 mmol, 0.062 g), HOBT (0.325 mmol, 0.043 g), DMAP (0.125 mmol, 0.015 g), pyridine (0.025 mmol, 2 µL) and cyclohexylmethanamine (0.25 mmol, 32 μL) in 8 mL DCM. The product was purified by column chromatography over silica gel and obtained as a yellowish solid with a yield of 72% (R*_f_* = 0.25 (S_2_)). LC/MS: C_20_H_27_N_3_O (95%) *m*/*z*: 326.22, found 326.05. ds: (**1RS**,**3SR**)-**5/**(**1RS**,**3RS**)-**5** = 10:4. ^1^H NMR (500 MHz, CDCl_3_) δ 8.05 (s, 1H), 7.49 (d, *J* = 7.5 Hz, 1H), 7.27 (s, 1H), 7.19–7.17 (m, 1H), 7.17–7.13 (m, 1H), 7.11–7.07 (m, 1H), 4.23–4.17 (m, 1H), 3.59 (dd, *J* = 4.6 Hz, 11.5 Hz, 1H), 3.33–3.27 (m, 1H), 3.22–3.11 (m, 2H), 2.71 (ddd, *J* = 2.6 Hz, 11.5 Hz, 15.8 Hz, 1H), 1.74 (dd, J = 3.4 Hz, 13.1 Hz, 5H), 1.51–1.46 (m, 3H), 1.29–1.12 (m, 4H), 1.01–0.91 (m, 2H) *NH* proton was not detected. ^13^C NMR (126 MHz, CDCl_3_) δ 172.9, 170.6, 137.0, 136.1, 127.4, 121.9, 119.1, 110.9, 108.7, 77.0, 58.1, 49.5, 45.5, 38.1, 31.0, 26.0, 20.4.

- *N*-(Adamantan-1-yl)-1-ethyl-2,3,4,9-tetrahydro-1*H*-pyrido[3,4-*b*]indole-3-carboxamide (**6**): (**1RS**,**3SR**)-**6/**(**1RS**,**3RS**)-**6**

Compound **6** was prepared using 1-ethyl-2,3,4,9-tetrahydro-1*H*-pyrido[3,4-*b*]indole-3-carboxylic acid (**1c**) (0.25 mmol, 0.061 g) and EDC (0.325 mmol, 0.062 g), HOBT (0.325 mmol, 0.037 g), DMAP (0.125 mmol, 0.015 g), pyridine (0.025 mmol, 2 µL) and adamantan-1-amine (0.25 mmol, 0.27 g) in 8 mL DCM. The product was purified by column chromatography over silica gel and obtained as a white solid with a yield of 60% (R*_f_* = 0.38 (S_4_)). LC/MS: C_24_H_31_N_3_O (96%) *m*/*z*: 378.25, found 378.30. ds: (**1RS**,**3SR**)-**6/**(**1RS**,**3RS**)-**6** = 11:4.4. ^1^H NMR (500 MHz, CDCl_3_) δ 8.62 (d, *J* = 1.7 Hz, 1H), 8.52 (s, 1H), 7.48–7.44 (m, 1H), 7.32–7.25 (m, 1H), 7.08–7.03 (m, 1H), 6.89 (s, 1H), 4.04 (d, *J* = 5.2 Hz, 1H), 3.44 (dd, *J* = 4.6 Hz, 10.9 Hz, 1H), 3.26 (ddd, *J* = 1.7 Hz, 4.4 Hz, 15.6 Hz, 1H), 2.67 (ddd, *J* = 2.3 Hz, 11.2 Hz, 15.8 Hz, 1H), 2.14–2.01 (m, 9H), 1.80 (ddd, *J* = 4.6 Hz, 7.4 Hz, 14.3 Hz, 1H), 1.74–1.62 (m, 7H), 1.03 (t, *J* = 7.2 Hz, 3H) *NH* indole was not detected. ^13^C NMR (126 MHz, CDCl_3_) δ 171.3, 135.0, 128.1, 120.9, 120.0, 119.0, 110.6, 107.1, 70.2, 56.3, 42.0, 36.1, 30.3, 27.2, 25.0, 11.1.

- *N*-(3,3-dimethylbutyl)-1-octyl-2,3,4,9-tetrahydro-1*H*-pyrido[3,4-*b*]-indole-3-carboxamide (**7**): (**1RS**,**3SR**)-**7/**(**1RS**,**3RS**)-**7**

Compound **7** was prepared using 1-octyl-2,3,4,9-tetrahydro-1*H*-pyrido[3,4-*b*]-indole-3-carboxylic acid (**1d**) (0.5 mmol, 0.145 g) and EDC (0.65 mmol, 0.124 g), HOBT (0.65 mmol, 0.086 g), DMAP (0.25 mmol, 0.30 g), pyridine (0.025 mmol, 2 µL) and 3,3-dimethylbutan-1-amine (0.5 mmol, 56 μL) in 8 mL DCM. The product was purified by column chromatography over silica gel (DCM/EtOAc = 9:1) and obtained as a dark yellow oil with a yield of 15% (R*_f_* = 0.22 (S_3_)). LC/MS: C_26_H_41_N_3_O (96%) *m*/*z* 412.32, found 412.28. ds: (**1RS**,**3SR**)-**7/**(**1RS**,**3RS**)-**7** = 10:4.7. ^1^H NMR (500 MHz, CDCl_3_) δ 7.85–7.70 (m, 1H), 7.52 (d, *J* = 7.7 Hz, 1H), 7.32 (dd, *J* = 8.0 Hz, 17.8 Hz, 1H), 7.19–7.14 (m, 1H), 7.17–7.08 (m, 1H), 7.05–7.00 (m, 1H), 4.14 (dd, *J* = 2.0 Hz, 4.9 Hz, 1H), 3.55 (dd, *J* = 4.3 Hz, 11.2 Hz, 1H), 3.40–3.30 (m, 3H), 2.70 (ddd, *J* = 2.6 Hz, 11.5 Hz, 15.5 Hz, 1H), 2.02–1.93 (m, 1H), 1.75 (q, *J* = 7.5 Hz, 1H), 1.67–1.43 (m, 4H), 1.39–1.25 (m, 8H), 1.00–0.95 (m, 9H), 0.90 (t, *J* = 6.7 Hz, 6H). ^13^C NMR (126 MHz, CDCl_3_) δ 135.9, 127.5, 121.9, 119.7, 118.4, 110.8, 109.5, 57.8, 53.8, 43.4, 35.9, 31.9, 29.5, 22.7, 14.2.

- 1-(4-chlorophenyl)-*N*-(3,3-dimethylbutyl)-2,3,4,9-tetrahydro-1*H*-pyrido[3,4-*b*]indole-3-carboxamide (**8**): (**1RS**,**3SR**)

Compound **8** was prepared using 1-(4-chlorophenyl)-2,3,4,9-tetrahydro-1*H*-pyrido[3,4-*b*]indole-3-carboxylic acid (**1e**) (0.25 mmol, 0.081 g) and EDC (0.325 mmol, 0.062 g), HOBT (0.325 mmol, 0.043 g), DMAP (0.125 mmol, 0.015 g), pyridine (0.1 mmol, 8 µL) and 3,3-dimethylbutan-1-amine (0.25 mmol, 33 μL) in 15 mL DCM. The product was purified by column chromatography over silica gel and obtained as an off-white solid with a yield of 22% (R*_f_* = 0.11 (S_3_)). LC/MS: C_24_H_28_ClN_3_O (97%) *m*/*z*: 410.20, found: 410.03. ^1^H NMR (500 MHz, CDCl_3_) δ 7.82 (s, 1H), 7.57 (d, *J* = 8.0 Hz, 1H), 7.30–7.24 (m, 4H), 7.18 (dd, *J* = 1.2 Hz, 8.0 Hz, 1H), 7.16–7.11 (m, 3H), 6.80–6.74 (m, 1H), 5.21 (s, 1H), 3.53–3.47 (m, 1H), 3.29 (s, 1H), 3.27–3.15 (m, 1H), 2.90–2.82 (m, 1H), 1.44–1.37 (m, 2H), 1.25 (d, *J* = 1.1 Hz, 2H), 0.91 (s, 9H). ^13^C NMR (126 MHz, CDCl_3_) δ 172.4, 139.1, 136.3, 134.2, 129.7, 127.1, 122.4, 119.9, 118.6, 110.7, 110.1, 60.5, 58.2, 43.3, 36.0, 29.8, 24.6, 21.0, 14.3.

- 1-(4-chlorophenyl)-*N*-(2,4,4-trimethylpentan-2-yl)-2,3,4,9-tetrahydro-1*H*-pyrido[3,4-*b*]indole-3-carboxamide (**9**): (**1RS**,**3SR**)

Compound **9** was prepared using 1-(4-chlorophenyl)-2,3,4,9-tetrahydro-1*H*-pyrido[3,4-*b*]indole-3-carboxylic acid (**1e**) (0.25 mmol, 0.081 g) and EDC (0.325 mmol, 0.062 g), HOBT (0.325 mmol, 0.043 g), DMAP (0.125 mmol, 0.015 g), pyridine (0.1 mmol, 8 µL) and 7,7-dimethyloctan-1-amine (0.25 mmol, 38 μL) in 15 mL DCM. The product was purified by column chromatography over silica gel (DCM/EtOAc = 14:1) and obtained as an off-white solid with a yield of 50% (R*_f_* = 0.33 (S_5_)). LC/MS: C_26_H_32_ClN_3_O (96%) *m*/*z*: 438.22 found 438.05. ^1^H NMR (500 MHz, CDCl_3_) δ 7.58 (s, 1H), 7.55–7.52 (m, 1H), 7.38–7.30 (m, 2H), 7.28–7.21 (m, 3H), 7.18–7.09 (m, 2H), 6.84 (s, 1H), 5.14 (t, *J* = 2.3 Hz, 1H), 3.58 (dd, *J* = 4.3 Hz, 11.1 Hz, 1H), 3.38–3.28 (m, 1H), 2.80–2.70 (m, 1H), 1.74 (s, 2H), 1.67–1.60 (m, 1H), 1.43 (s, 6H), 1.00 (s, 9H). ^13^C NMR (126 MHz, CDCl_3_) δ 171.4, 139.4, 136.3, 134.0, 129.6, 127.2, 122.3, 119.2, 110.8, 58.2, 52.2, 26.1.

- 1-(4-chlorophenyl)-*N*-((tetrahydrofuran-2-yl)methyl)-2,3,4,9-tetrahydro-1*H*-pyrido[3,4-*b*]indole-3-carboxamide (**10**): (**1RS**,**3SR**)

Compound **10** was prepared using 1-(4-chlorophenyl)-2,3,4,9-tetrahydro-1*H*-pyrido[3,4-*b*]indole-3-carboxylic acid (**1e**) (2.3 mmol, 0.76 g) and EDC (3.0 mmol, 0.58 g), HOBT (3.0 mmol, 0.4 g), DMAP (1.17 mmol, 0.143 g), pyridine (0.1mmol, 8 µL) and (tetrahydrofuran-2-yl)methanamine (2.3 mmol, 0.24 g) in 15 mL DCM. The product was purified by crystallization in hexane and a few drops acetone and obtained as an off-white solid with a yield of 35% (R*_f_* = 0.09 (S_3_)). LC/MS: C_23_H_24_ClN_3_O_2_ (95%) *m*/*z*: 410.16, found: 410.10. ^1^H NMR (500, DMSO-*d*_6_) δ ppm: 10.75 (d, *J* = 3.0 Hz, 1H), 7.97–7.87 (m, 1H), 7.45–7.38 (m, 1H), 7.37–7.28 (m, 2H), 7.25–7.18 (m, 2H), 7.21 (t, *J* = 7.4 Hz, 1H), 7.01 (t, *J* = 6.7 Hz, 1H), 6.97–6.90 (m, 1H), 5.18 (br. s, 1H), 3.85–3.50 (m, 3H), 3.38–2.99 (m, 4H), 2.90 (dd, *J* = 4.6 Hz, *J* = 15.1 Hz, 1H), 2.77–2.56 (m, 1H), 1.85–1.64 (m, 3H), 1.54–1.29 (m, 1H). ^13^CNMR (126 MHz, DMSO-*d*_6_) δ ppm: 172.7, 142.0, 136.1, 131.6, 130.2, 128.0, 128.0, 126.7, 120.9, 118.4, 117.7, 111.1, 108.1, 108.1, 77.1, 77.0, 67.2, 67.2, 53.2, 42.5, 42.4, 28.5, 28.4, 25.2, 25.2.

- *N*-((1,4-dioxan-2-yl)methyl)-1-(4-chlorophenyl)-2,3,4,9-tetrahydro-1*H*-pyrido[3,4-*b*]indole-3-carboxamide (**11**): (**1RS**,**3SR**)

Compound **11** was prepared using 1-(4-chlorophenyl)-2,3,4,9-tetrahydro-1*H*-pyrido[3,4-*b*]indole-3-carboxylic acid (**1e**) (2.3 mmol, 0.76 g) and EDC (3.0 mmol, 0.58 g), HOBT (3.0 mmol, 0.4 g), DMAP (1.17 mmol, 0.143 g), pyridine (0.1 mmol, 8 µL) and (1,4-dioxan-2-yl)methanamine (2.3 mmol, 0.27 g) in 15 mL DCM. The product was purified by crystallization in hexane and obtained as a white solid with a yield of 15% (R*_f_* = 0.07 (S_3_)). LC/MS: C_23_H_24_ClN_3_O_3_ (98%) *m*/*z*: 426.15, found 426.04.^1^H NMR (500, DMSO-*d*_6_) δ ppm: 10.72 (s, 1H), 7.99–7.85 (m, 1H), 7.44–7.40 (m, 1H), 7.38–7.33 (m, 2H), 7.26–7.17 (m, 3H), 7.04–6.90 (m, 2H), 5.18 (br.s., 1H), 3.71–3.46 (m, 5H), 3.43–3.35 (m, 2H), 3.19–3.02 (m, 4H), 2.90 (dd, *J* = 15.5 Hz, 1H), 2.76–2.61 (m, 1H). ^13^CNMR (126 MHz, DMSO-*d*_6_) δ ppm: 172.9, 142.0, 136.1, 134.0, 131.7, 130.2, 128.1, 126.7, 121.0, 118.4, 117.7, 111.1, 108.0, 73.6, 73.6, 68.7, 65.9, 65.9, 65.8, 53.2, 51.7, 25.0.

- 1-(4-chlorophenyl)-*N*-(3-morpholinopropyl)-2,3,4,9-tetrahydro-1*H*-pyrido[3,4-*b*]indole-3-carboxamide (**12**): (**1RS**,**3SR**)-**12/**(**1RS**,**3RS**)-**12**

Compound **12** was prepared using 1-(4-chlorophenyl)-2,3,4,9-tetrahydro-1*H*-pyrido[3,4-*b*]indole-3-carboxylic acid (**1e**) (1.2 mmol, 0.39 g) and EDC (1.5 mmol, 0.30 g), HOBT (1.5 mmol, 0.20 g), DMAP (0.6 mmol, 0.07 g), pyridine (0.05 mmol, 4 µL) and (1.2 mmol, 175 µL) in 10 mL DCM. The obtained crude product was purified by column chromatography over silica gel (DCM/methanol = 95:5) and obtained as pink needlelike crystals with a yield of 45% (R*_f_* = 0.1 (S_2_)). LC/MS: C_25_H_29_ClN_4_O_2_ (96%) *m*/*z*: 453.20, found 453.04. ds: (**1RS**,**3SR**)-**12/**(**1RS**,*RS*)-**12**= 10:1.5. ^1^H NMR (500, CDCl_3_) δ ppm: 8.00–7.89 (m, 1H), 7.70–7.58 (m, 2H), 7.39–7.34 (m, 3H), 7.30–7.19 (m, 4H), 5.34 (s, 1H), 3.68 (t, *J* = 4.0 Hz, 4H), 3.56 (dd, *J* = 10.5 Hz, 1H), 3.50–3.33 (m, 3H), 3.03–2.92 (m, 1H), 2.56–2.44 (m, 6H), 1.76 (quin, *J* = 6.5 Hz, 2H), *NH* proton was not detected. ^13^CNMR (126 MHz, CDCl_3_) δ ppm: 172.7, 139.9, 136.2, 133.9, 132.5, 129.9, 129.2, 128.7, 126.9, 122.3, 119.8, 118.5, 110.9, 110.4, 66.7, 66.7, 66.7, 57.3, 54.8, 53.8, 53.6, 52.4, 25.3, 25.0.

- 1-(4-chlorophenyl)-*N*-cyclohexyl-2,3,4,9-tetrahydro-1*H*-pyrido[3,4-*b*]indole-3-carboxamide (**13**)

Compound **13** was prepared using 1-(4-chlorophenyl)-2,3,4,9-tetrahydro-1*H*-pyrido[3,4-*b*]indole-3-carboxylic acid (**1e**) (0.25 mmol, 0.081 g) and EDC (0.325 mmol, 0.062 g), HOBT (0.325 mmol, 0.043 g), DMAP (0.125 mmol, 0.015 g), pyridine (0.1 mmol, 8 µL) and cyclohexanamine (0.25 mmol, 28 μL) in 4 mL DCM. The obtained product (mixture of *cis* and *trans* isomers) was purified and diastoreoisomers were separated by column chromatography over silica gel (R*_f_* = 0.3 and R*_f_* = 0.29 (S_5_), overall yield 67%. LC/MS: C_24_H_26_ClN_3_O (96%) *m*/*z*: 408.94, found: 409.01.

- (**1RS**,**3SR**)-1-(4-chlorophenyl)-*N*-cyclohexyl-2,3,4,9-tetrahydro-1*H*-pyrido[3,4-*b*]indole-3-carboxamide (*trans*-**13**)

^1^H NMR (500 MHz, CDCl_3_) δ 7.94 (s, 1H), 7.57 (d, *J* = 7.4 Hz, 1H), 7.32–7.28 (m, 3H), 7.20 (dt, *J* = 1.1 Hz, 7.4 Hz, 1H), 7.17–7.13 (m, 3H), 6.75 (d, *J* = 8.6 Hz, 1H), 5.19 (s, 1H), 3.78–3.70 (m, 1H), 3.52 (dd, *J* = 4.6 Hz, 9.7 Hz, 1H), 3.28 (dd, *J* = 4.9 Hz, 15.8 Hz, 1H), 2.88 (ddd, *J* = 1.7 Hz, 9.9 Hz, 15.9 Hz, 1H), 2.10–1.99 (br.s., 1H), 1.93–1.83 (m, 2H), 1.70 (tdd, *J* = 4.1 Hz, 8.6 Hz, 16.5 Hz, 2H), 1.62 (td, *J* = 3.7 Hz, 12.6 Hz, 1H), 1.43–1.30 (m, 2H), 1.25–1.08 (m, 3H) ^13^C NMR (126 MHz, CDCl_3_) δ 171.6, 139.8, 136.2, 133.8, 132.5, 129.9, 128.7, 127.0, 122.3, 119.7, 118.5, 110.9, 110.5, 54.7, 52.3, 47.7, 33.1, 32.9, 25.5, 24.8, 24.7.

- (**1SR**,**3SR**)-1-(4-chlorophenyl)-*N*-cyclohexyl-2,3,4,9-tetrahydro-1*H*-pyrido[3,4-*b*]indole-3-carboxamide (*cis*-**13**)

^1^H NMR (500 MHz, CDCl_3_) δ 7.58–7.52 (m, 2H), 7.37–7.33 (m, 2H), 7.26–7.24 (m, 2H), 7.23–7.20 (m, 1H), 7.17–7.09 (m, 2H), 6.74 (d, *J* = 8.6 Hz, 1H), 5.15 (t, *J* = 2.3 Hz, 1H), 3.84–3.74 (m, 1H), 3.66 (dd, *J* = 4.3 Hz, 11.1 Hz, 1H), 3.33 (ddd, *J* = 1.7 Hz, 4.4 Hz, 15.6 Hz, 1H), 2.82 (ddd, *J* = 2.3 Hz, 11.1 Hz, 15.8 Hz, 1H), 1.96–1.85 (m, 2H), 1.75–1.65 (m, 2H), 1.61 (td, *J* = 3.7 Hz, 13.1 Hz, 1H), 1.41–1.29 (m, 2H), 1.22–1.09 (m, 3H).

- 1-(4-chlorophenyl)-*N*-(cyclohexylmethyl)-2,3,4,9-tetrahydro-1*H*-pyrido[3,4-*b*]indole-3-carboxamide (**14**): (**1RS**,**3SR**)

Compound **14** was prepared using 1-(4-chlorophenyl)-2,3,4,9-tetrahydro-1*H*-pyrido[3,4-*b*]indole-3-carboxylic acid (**1e**) (0.25 mmol, 0.081 g) and EDC (0.325 mmol, 0.062 g), HOBT (0.325 mmol, 0.043 g), DMAP (0.125 mmol, 0.015 g), pyridine (0.1 mmol, 8 µL) and cyclohexylmethanamine (0.25 mmol, 32 μL) in 8 mL DCM. The product was purified by crystallization in hexane and a few drops of acetone to give a pale yellow solid with a yield of 25% (R*_f_* = 0.09 (S_3_)). LC/MS: C_25_H_28_ClN_3_O (96%) *m*/*z*: 422.19 found: 422.10. ^1^H NMR (500 MHz, CDCl_3_) δ 7.76 (s, 1H), 7.57 (d, *J* = 8.0 Hz, 1H), 7.30–7.26 (m, 3H), 7.21–7.17 (m, 1H), 7.17–7.12 (m, 3H), 6.91 (t, *J* = 5.7 Hz, 1H), 5.23 (s, 1H), 3.56 (dd, *J* = 4.6 Hz, 9.7 Hz, 1H), 3.31 (dd, *J* = 5.1 Hz, 16.0 Hz, 1H), 3.16–3.09 (m, 1H), 3.02 (td, *J* = 6.4 Hz, 13.5 Hz, 1H), 2.89 (ddd, *J* = 1.4 Hz, 10.0 Hz, 16.0 Hz, 1H), 1.74–1.62 (m, 5H), 1.44 (ttd, *J* = 3.4 Hz, 7.3 Hz, 14.5 Hz, 1H), 1.29–1.07 (m, 4H), 0.95–0.85 (m, 2H). ^13^C NMR (126 MHz, CDCl_3_) δ 172.5, 139.3, 136.3, 134.6, 134.0, 130.0, 129.3, 127.2, 122.3, 119.9, 118.6, 111.1, 110.4, 58.4, 45.5, 38.0, 31.0, 26.5, 25.9.

### 3.3. PKa and Log D Calculation

The decimal logarithm distribution coefficient (Log D) was calculated using the MarvinSketch software (v. 20.12.0, ChemAxon Ltd., Cambridge, MA, USA).

### 3.4. P. falciparum Cultures and Drug Susceptibility Assay

*Plasmodium falciparum* cultures were established according to the method of Trager and Jensen, with slight modifications [24]. The CQ-susceptible strain, D10, and the CQ-resistant strain, W2, were maintained in human type A-positive red blood cells at 5% hematocrit in RPMI 1640 medium with the addition of 1% AlbuMax, 0.01% hypoxanthine, 20 mM Hepes, and 2 mM glutamine. Cultures were maintained at 37 °C in a gas mixture consisting of 1% O_2_, 5% CO_2_, and 94% N_2_. Compounds were dissolved in DMSO and diluted with medium to achieve the required concentrations (final DMSO concentration <1%, which is non-toxic to the parasite). Drugs were introduced into 96-well flat-bottomed microplates and serial dilutions made. Asynchronous cultures with parasitaemia of 1–1.5% and 1% final hematocrit were aliquoted into the plates and incubated for 72 h at 37 °C. Parasite growth was determined spectrophotometrically (OD_650_) by measuring the activity of parasite lactate dehydrogenase (pLDH), according to a modified version of the method of Makler, in both control and drug-treated cultures [25]. The antimalarial activity is expressed as 50% inhibitory concentrations (IC_50_); each IC_50_ value is the mean of at least three separate experiments performed in duplicate.

### 3.5. Cytotoxicity Assay on HMEC-1 Cell Line

The long-term human microvascular endothelial cell line (HMEC-1) was maintained in MCDB 131 medium supplemented with 10% fetal calf serum, 10 ng/mL of epidermal growth factor, 1 µg/mL of hydrocortisone, 2 mM glutamine and 20 mM Hepes buffer. For the cytotoxicity assays, cells were treated with serial dilutions of test compounds and cell proliferation evaluated using the MTT assay. Plates were incubated for 72 h at 37 °C in 5% CO_2_, then 20 µL of a 5 mg/mL solution of 3-(4,5-dimethylthiazol-2-yl)-2,5-diphenyltetrazolium bromide (MTT) in PBS was added for an additional 3 h at 37 °C. The plates were then centrifuged, the supernatants discarded, and the dark blue formazan crystals dissolved using 100 µL of lysing buffer consisting of 20% (*w/v*) of a solution of SDS, 40% of *N*,*N* dimethylformamide in H_2_O, at pH 4.7 adjusted with 80% acetic acid. The plates were then read on a microplate reader (Synergy 4 Bio-Tek Instruments, Thermo Fisher Scientific, Waltham, MA, USA) at a test wavelength of 550 nm and a reference wavelength of 650 nm. All the tests were performed in triplicate at least three times.

### 3.6. Determination of Hemolytic Activity

The study protocol was approved by the Bioethics Commission at the Lower Silesian Medical Chamber (1/PNHAB/2018, approval date 14 February 2018), and experiments were performed as previously described [26]. Compounds dissolved in DMSO were added in a volume corresponding to a final concentration of 10 µM in the sample. Negative (erythrocytes in PBS buffer), positive (erythrocytes in distilled water), and DMSO controls were also prepared.

### 3.7. Molecular Modeling

Molecular modeling studies were performed to evaluate the possible mechanism of action of compound (1*S*,3*R*)-**7**. Crystal structures of five enzymes, whose activities are essential for the functioning of *P. falciparum*, were taken from the Protein Data Bank. These enzymes were: phosphoethanoloamine methyltransferase (PMT—code 3UJ9), falcipain-2 (FP2—code 3BPF), falcipain-3 (FP3—code 3BPM), lactate dehydrogenase (LDH—code 1T26), and malate dehydrogenase (MDH—code 6R8G). All simulations were performed using the Small-Molecule Drug Discovery Suite (Schrödinger, Inc, New York, NY, USA). The crystal structure of each enzyme was refined using the Protein Preparation Wizard [27]: system pH was set at 7.4 ± 0.2, all co-crystallized molecules (water molecules, other solvents using in the crystallization process), except a ligand (product/inhibitor/cofactor), were removed, hydrogen atoms were added, and the energy of the whole system was minimized using an OPLS3e force field. The structure of compound (1*S*,3*R*)-**7** was optimized using the LigPrep tool (Schrödinger, Inc, New York, NY, USA). The proper protonation state at pH 7.4 was additionally verified using MarvinSketch software (ChemAxon, chemaxon.com accessed on 1st of April 2021, Cambridge, MA, USA). Induced-Fit Docking (IFD) was applied to dock compound (1*S*,3*R*)-**7** to the selected enzyme crystal structures and its binding mode within each of the enzymes was evaluated [28]. For each crystal structure, the box centroid was set on the originally co-crystallized ligand: PMT—phosphocholine (product of the enzymatic reaction performed by PMT); FP2—epoxysuccinate (inhibitor); FP3—leupeptin (inhibitor); LDH—1,4-fihydronicotinamide adenine dinucleotide (NADH, cofactor); MDH—nicotinamide adenine dinucleotide (NAD, cofactor). No constraints were applied during IFD. Target-ligand complexes, obtained as an IFD output, were analyzed based on the observed molecular interactions. The observed binding modes of compound (1*S*,3*R*)-**7** were compared with those captured for the ligands in the original crystal structures.

## 4. Conclusions

Malaria is still a major life-threatening infectious disease and, unfortunately, drug resistance to commonly used antimalarial drugs has become a serious problem. In this context, the search for new effective and selective agents active against this neglected parasite is obvious. Our studies led to the identification of a promising lead compound among a series of tetrahydro-β-carbolines that were designed and tested. Compound **7** has the highest activity against *P. falciparum* and is without any toxicity at the tested dose of 10 µM. Moreover, compound (1*S*,3*R*)-**7** has structural features enabling it to interact with enzymes essential for the functioning of *P. falciparum,* but a thorough explanation of its mechanism of action as well as the optimal delivery system are still required. Interestingly, the chemical structure of compound (1*S*,3*R*)-**7** also appears to aid in its stabilization within the protein crystal co-structures. Our results also provide a basis for further scaffold optimization, that could lead to the design, synthesis, and identification of additional compounds with improved antiplasmodial potency.

## Figures and Tables

**Table 1:** Prediction of Log D for compounds **2**–**14** and CQ.

Comp	Structure	Log D			Comp	Structure	Log D		
		pH 7.4	pH 7.2	pH 5.5			pH 7.4	pH 7.2	pH 5.5
**2**	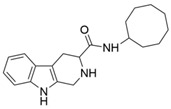	2.53	2.40	0.87	**10**	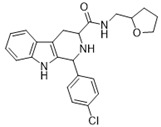	3.39	3.34	2.12
**3**	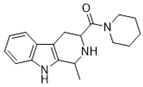	1.73	1.59	0.05	**11**	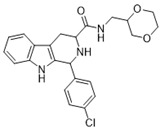	2.77	2.71	1.50
**4**	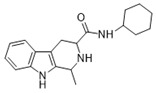	2.47	2.33	0.80	**12**	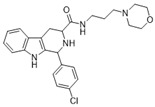	2.69	2.53	0.05
**5**	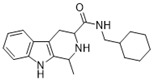	2.79	2.65	1.11	**1RS**,**3RS-13**	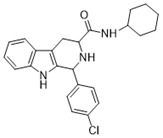	4.47	4.72	3.50
**6**	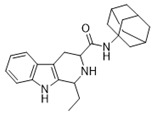	3.18	3.03	1.47	**1RS**,**3SR-13**	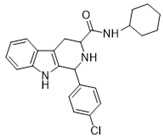	4.47	4.72	3.50
**7**	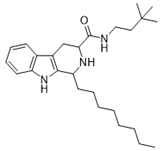	5.65	5.49	3.93	14	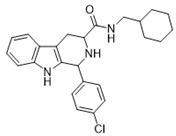	5.09	5.03	3.82
**8**	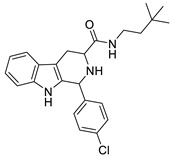	4.88	4.83	3.61	CQ	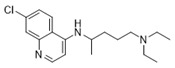	0.88	0.64	0.76
**9**	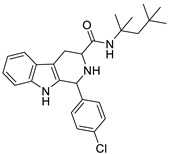	5.58	5.53	4.31					

**Table 2:** The antiplasmodial activity of compounds (**2**–**14**) against the D10 (CQ-sensitive) and W2 (CQ-resistant) strains of *P. falciparum* and relevant RI.

Compound	*P. falciparum* IC_50_ (µM)		RI ^a^
	D10	W2	
**2**	21.87 ± 5.82	10.12 ± 2.14	0.46
**3**	13.17 ± 2.85	9.60 ± 0.78	0.73
**4**	21.38 ± 4.70	20.10 ± 2.79	0.94
**5**	33.47 ± 7.58	16.19 ± 4.11	0.48
**6**	9.73 ± 0.96	11.36 ± 0.82	1.17
**7**	4.45 ± 0.83	4.00 ± 0.53	0.90
**8**	10.48 ± 1.53	7.80 ± 1.54	0.74
**9**	8.41 ± 1.20	6.41 ± 1.05	0.76
**10**	29.36 ± 7.88	20.54 ± 2.20	0.70
**11**	35.36 ± 4.86	29.07 ± 4.05	0.82
**12**	11.45 ± 0.34	6.15 ± 0.46	0.54
**1RS**,**3RS-13**	16.05 ± 3.36	13.46 ± 1.99	0.84
**1RS**,**3SR-13**	12.53 ± 0.71	9.13 ± 0.83	0.73
**14**	- ^b^	- ^b^	-
CQ	0.017 ± 0.006	0.27 ± 0.07	15.88

^a^ RI = IC_50_ CQ resistant *P. falciparum* strain/IC_50_ CQ sensitive *P. falciparum* strain; ^b^ IC_50_ > 47.40 µM.

**Table 3:** Cytotoxicity of compounds **3**, **6**, **7**, **8**, **9**, **12**, **1RS**,**3RS-13**, **1RS**,**3SR-13**, and CQ on the HMEC-1 cell line and relevant selectivity indexes (SI).

Compound	IC_50_ (µM)	SI ^a^	
		D10	W2
**3**	157.18 ± 42.50	11.93	16.37
**6**	21.09 ± 8.82	2.17	1.86
**7**	72.02 ± 22.40	16.18	18.00
**8**	17.95 ± 9.46	1.71	2.30
**9**	18.93 ± 10.16	2.25	2.95
**12**	102.07 ± 14.83	8.91	16.60
**1RS**,**3RS-13**	53.78 ± 21.89	3.35	4.00
**1RS**,**3SR-13**	- ^b^	-	-
CQ	>38 ^c^	-	-

^a^ SI = IC_50_ HMEC-1/IC_50_ *P. falciparum* strain; ^b^ IC_50_ > 122.57 µM; ^c^ data from [21].

## Data Availability

The data presented in this study are available in article or Supplementary Materials.

## Supplementary Materials

The following are available online at https://www.mdpi.com/article/10.3390/ijms222413569/s1.
