# Supplementary material for: Synthesis, Molecular Docking and Antiplasmodial Activities of New Tetrahydro-β-Carbolines
Source: Int J Mol Sci. 2021 Dec 17;22(24):13569. doi: 10.3390/ijms222413569 (PMC8707145; doi:10.3390/ijms222413569)
Supplement: Supplementary file 1 [file ijms-22-13569-s001.zip › ijms-1476685- supplementary final-done.pdf]

International Journal of *Molecular Sciences*

MDPI

Supplementary Materials

Supplementary Table S1. ^{1}H- and ^{13}C-NMR spectra for compounds **5**,**7**,**9**–**14** provided.

**Comp-dound**	**^{1}H-NMR**
**2**	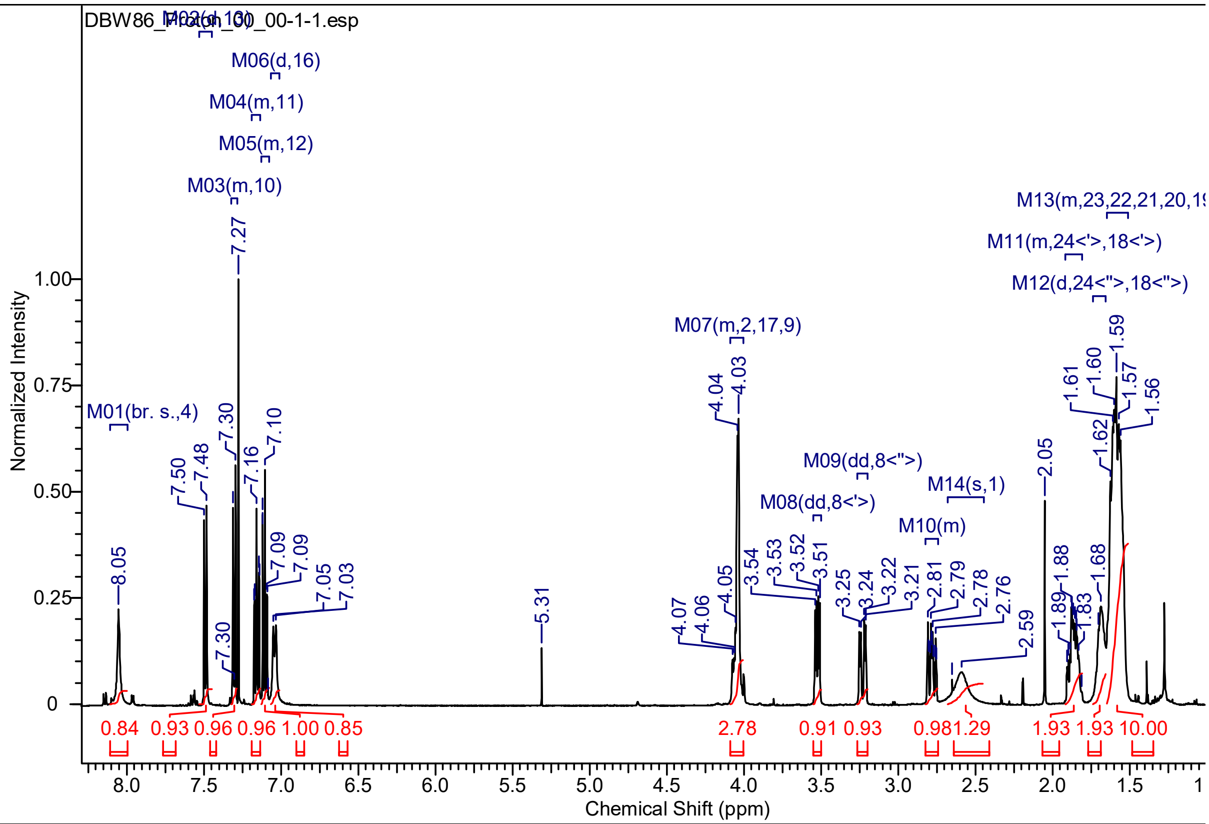

3

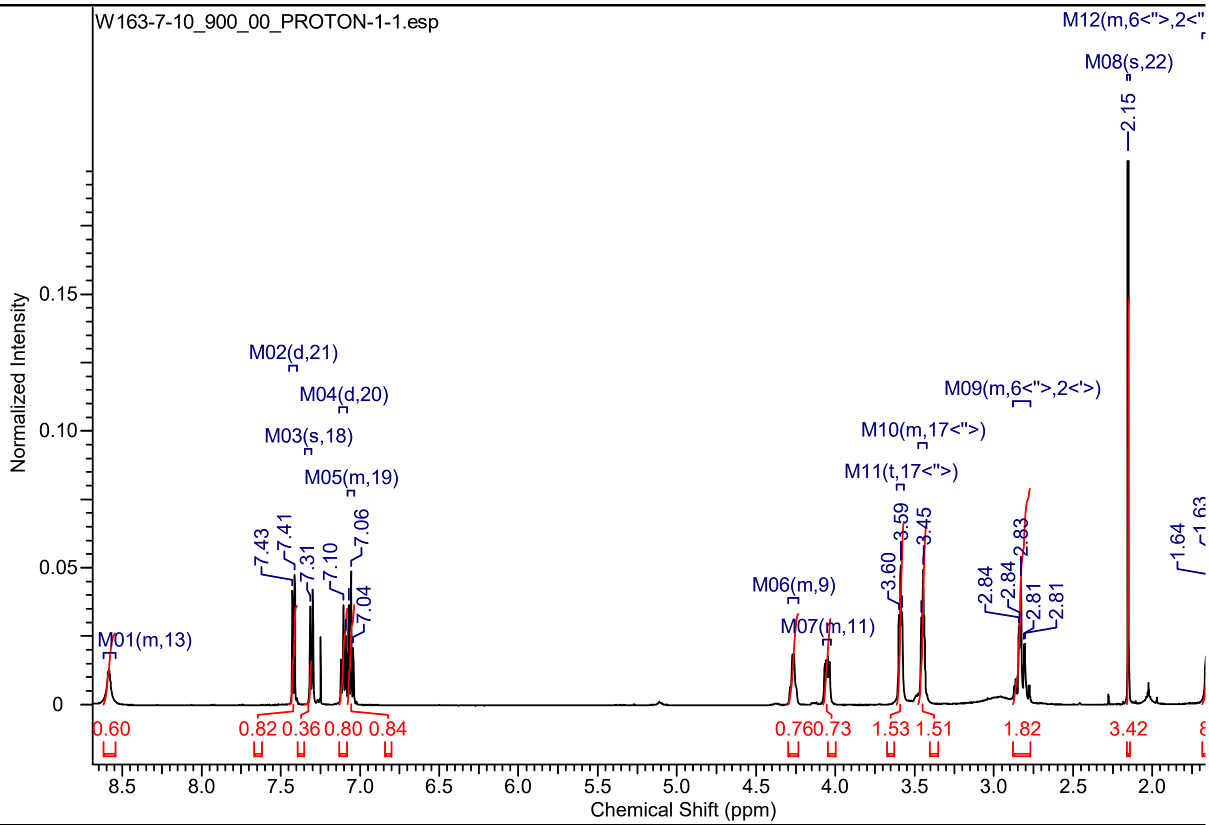
W163-7-10_900_00_PROTON-1-1.esp
Normalized Intensity
Chemical Shift (ppm)
M01(m,13)
M02(d,21)
M03(s,18)
M04(d,20)
M05(m,19)
M06(m,9)
M07(m,11)
M08(s,22)
M09(m,6<">,2<'>)
M10(m,17<">)
M11(t,17<">)
M12(m,6<">,2<"

4

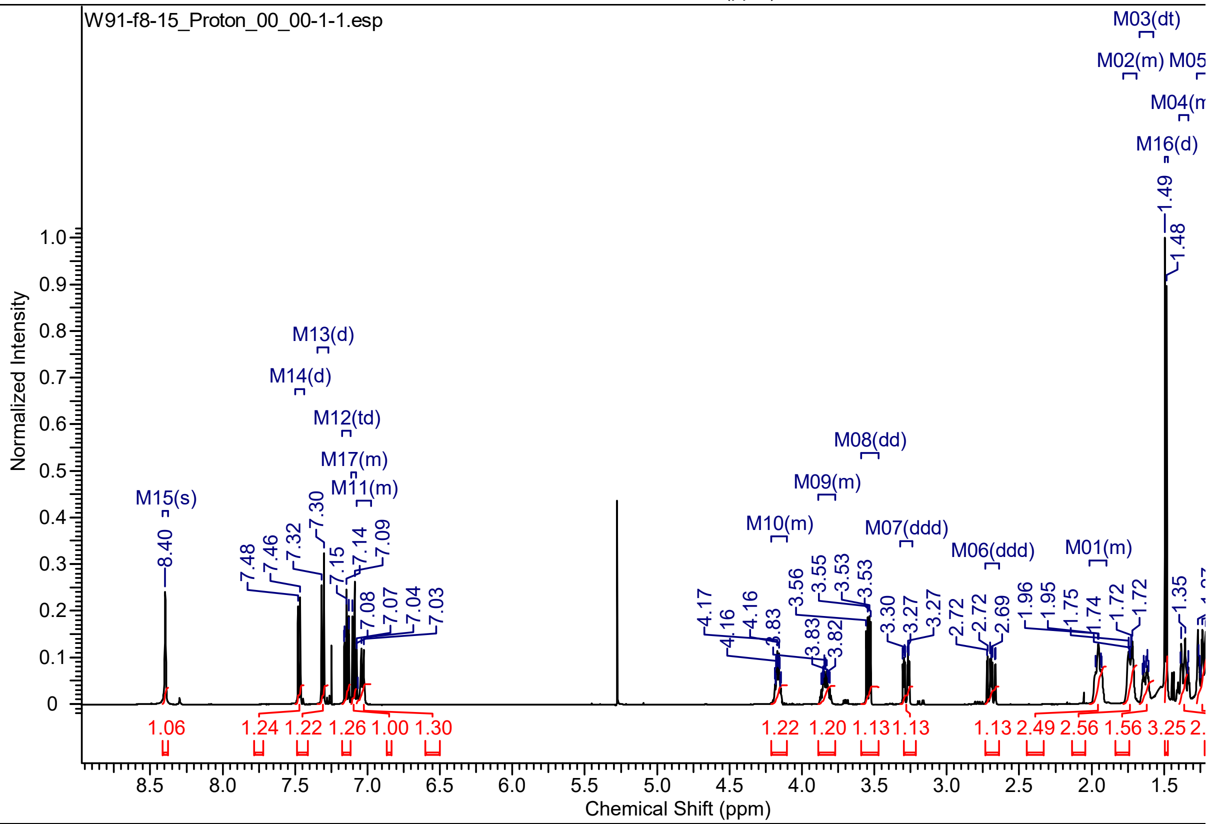
W91-f8-15_Proton_00_00-1-1.esp
Normalized Intensity
Chemical Shift (ppm)
M01(m)
M02(m)
M03(dt)
M06(ddd)
M07(ddd)
M08(dd)
M09(m)
M10(m)
M11(m)
M12(td)
M13(d)
M14(d)
M15(s)
M16(d)
M17(m)

5

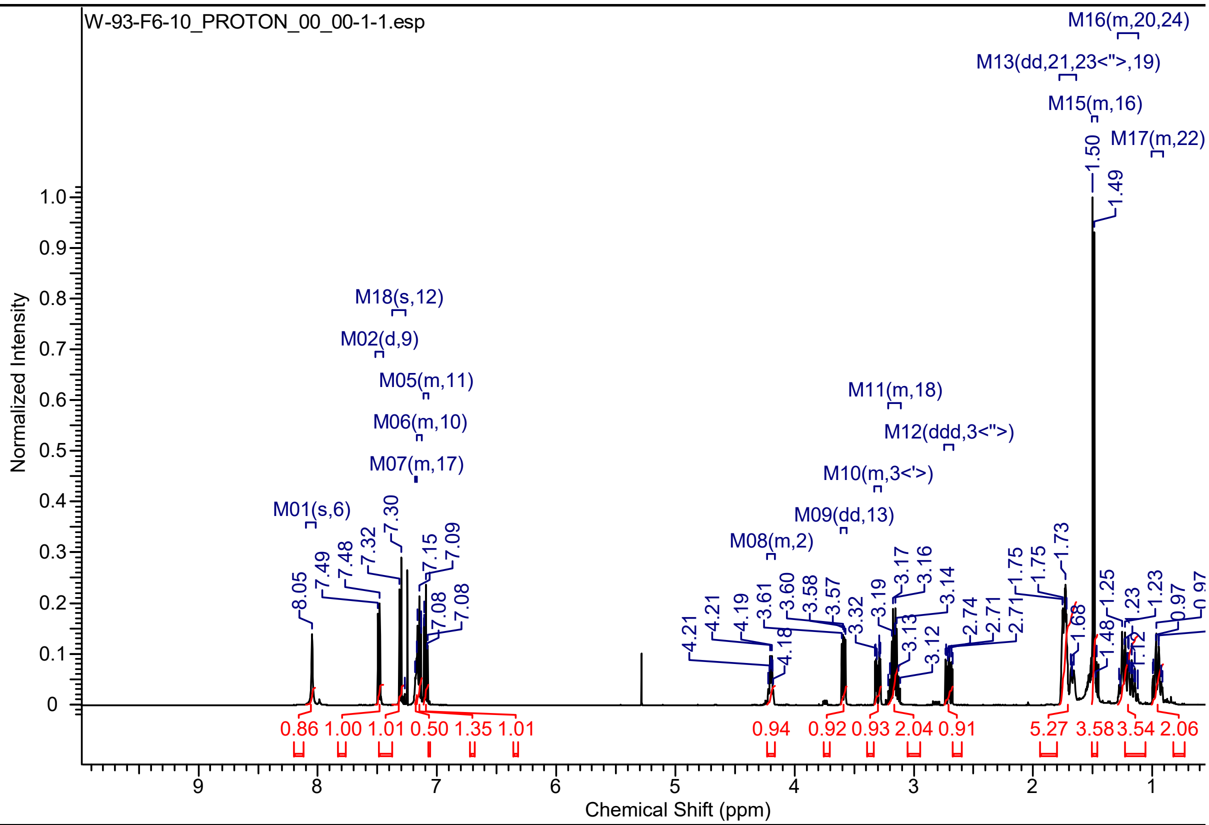
W-93-F6-10_PROTON_00_00-1-1.esp
Normalized Intensity
Chemical Shift (ppm)
M16(m,20,24)
M13(dd,21,23<''>,19)
M15(m,16)
M17(m,22)
M18(s,12)
M02(d,9)
M05(m,11)
M06(m,10)
M07(m,17)
M01(s,6)
M11(m,18)
M12(ddd,3<''>)
M10(m,3<'>)
M09(dd,13)
M08(m,2)
0.86 1.00 1.01 0.50 1.35 1.01
0.94 0.92 0.93 2.04 0.91
5.27 3.58 3.54 2.06

6

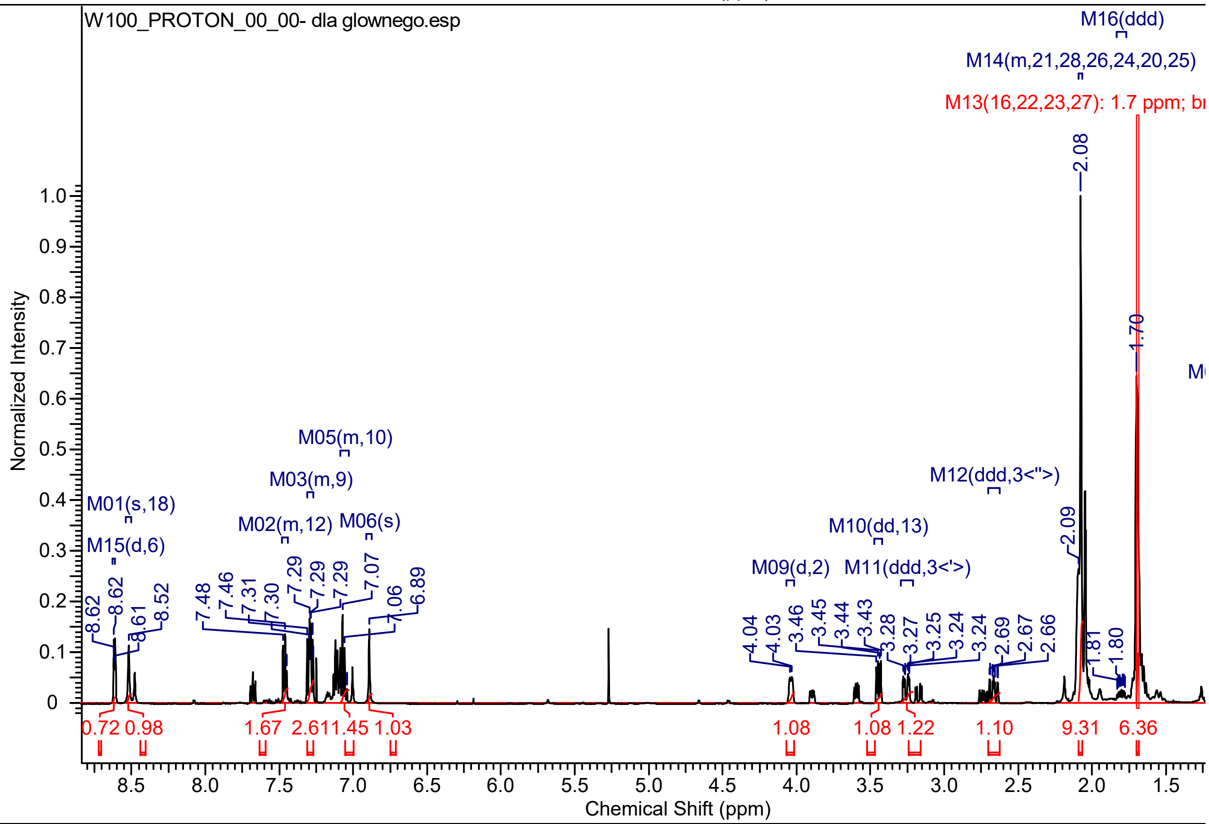
W100_PROTON_00_00- dla glownego.esp
Normalized Intensity
Chemical Shift (ppm)
M16(ddd)
M14(m,21,28,26,24,20,25)
M13(16,22,23,27): 1.7 ppm; br
M05(m,10)
M03(m,9)
M01(s,18)
M02(m,12)
M06(s)
M15(d,6)
M12(ddd,3<''>)
M10(dd,13)
M09(d,2)
M11(ddd,3<'>)
0.72 0.98
1.67 2.61 1.45 1.03
1.08 1.08 1.22 1.10 9.31 6.36

7

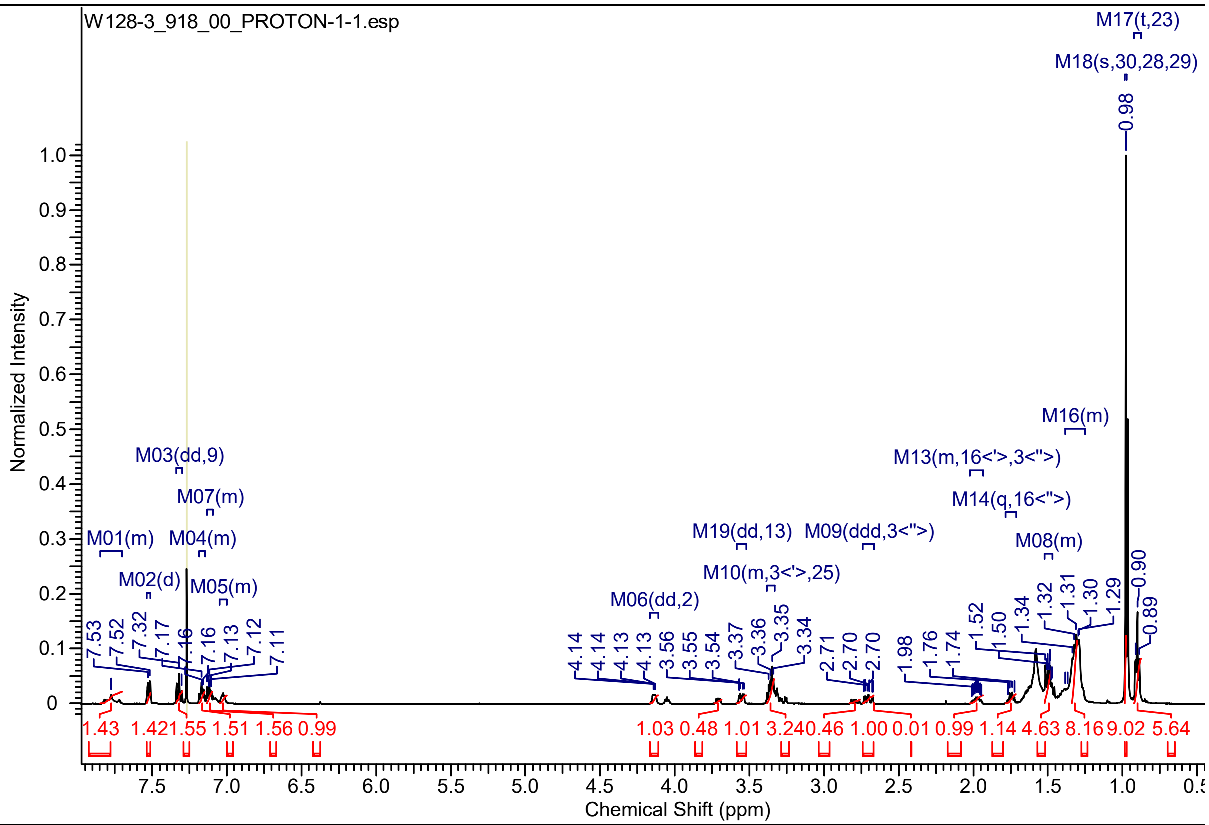
W128-3_918_00_PROTON-1-1.esp
Normalized Intensity
Chemical Shift (ppm)

8

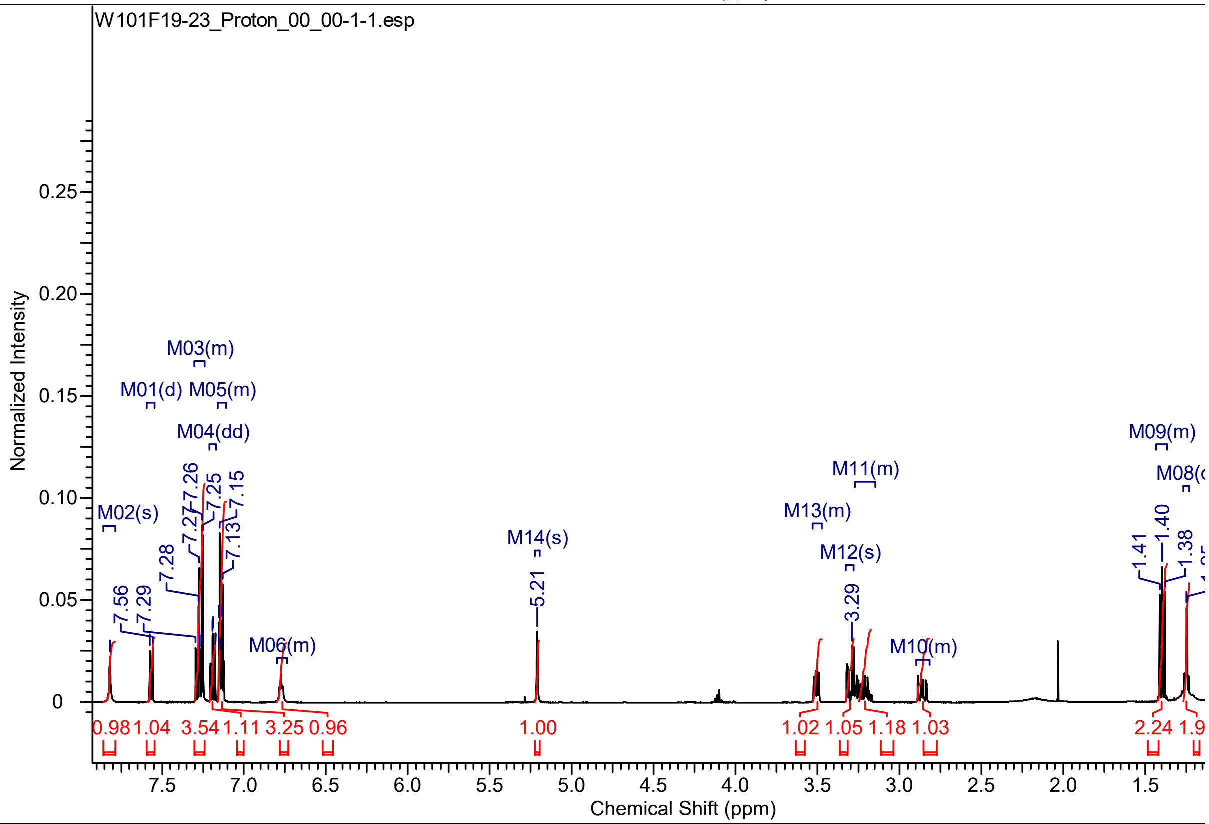
W101F19-23_Proton_00_00-1-1.esp
Normalized Intensity
Chemical Shift (ppm)

**9**	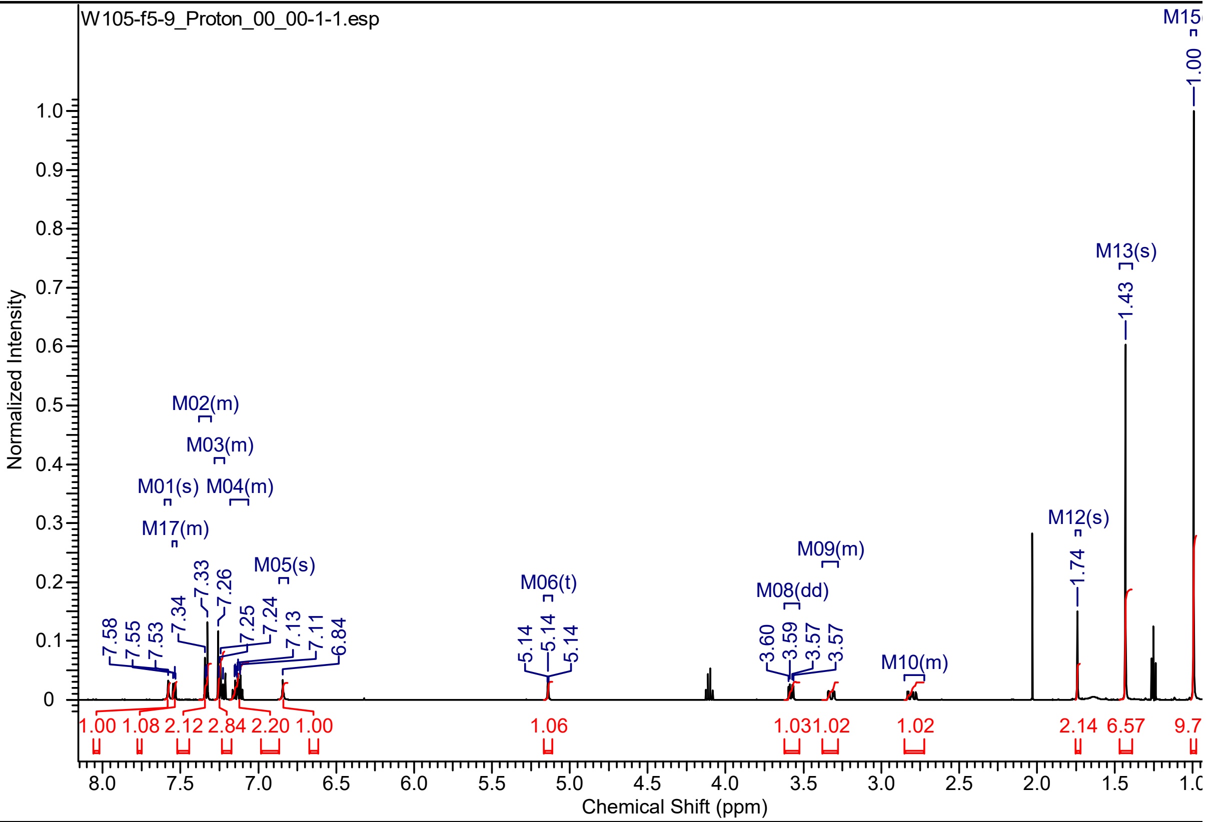
**10**	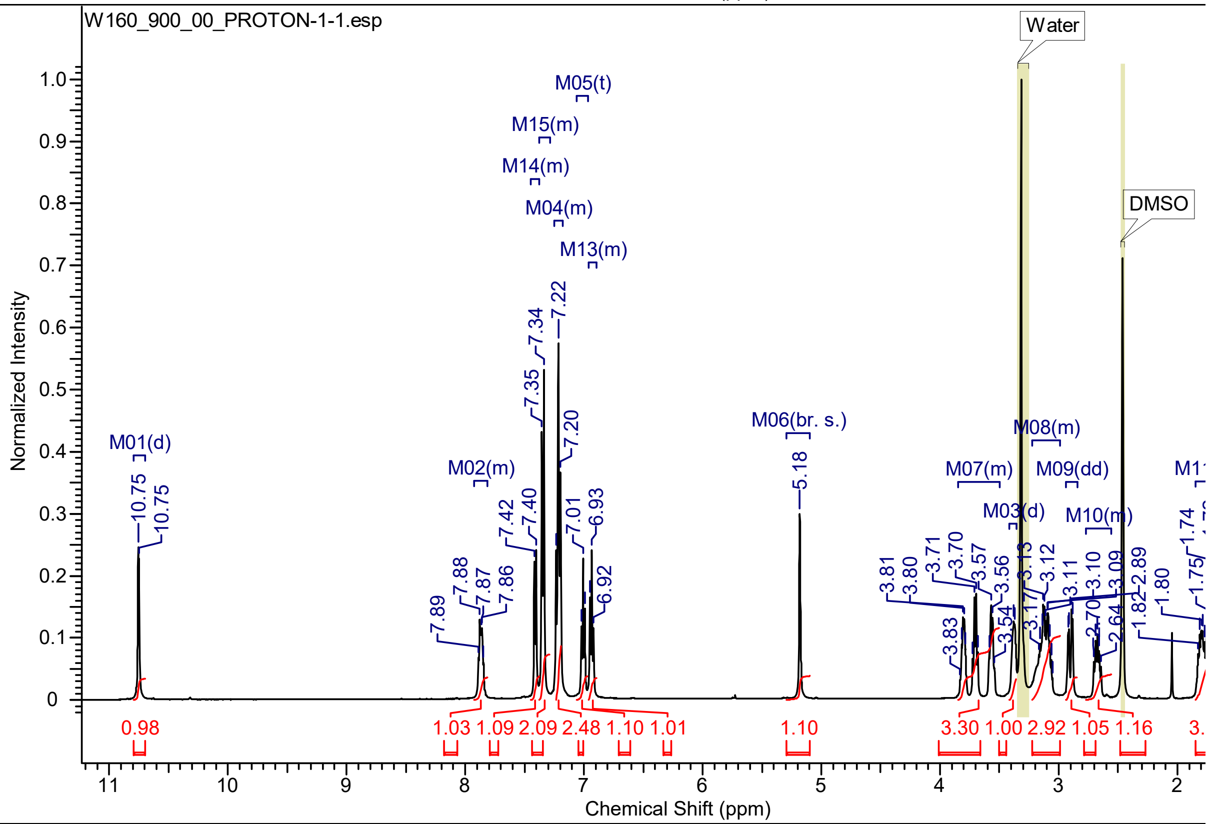

W105-f5-9_Proton_00_00-1-1.esp
Normalized Intensity
Chemical Shift (ppm)
M01(s)
M02(m)
M03(m)
M04(m)
M05(s)
M06(t)
M08(dd)
M09(m)
M10(m)
M12(s)
M13(s)
M15
M17(m)
1.00 1.08 2.12 2.84 2.20 1.00
1.06
1.03 1.02
1.02
2.14 6.57

W160_900_00_PROTON-1-1.esp
Water
DMSO
Normalized Intensity
Chemical Shift (ppm)
M01(d)
M02(m)
M03(d)
M04(m)
M05(t)
M06(br. s.)
M07(m)
M08(m)
M09(dd)
M10(m)
M13(m)
M14(m)
M15(m)
0.98
1.03 1.09 2.09 2.48 1.10 1.01
1.10
3.30 1.00 2.92 1.05 1.16

**11**	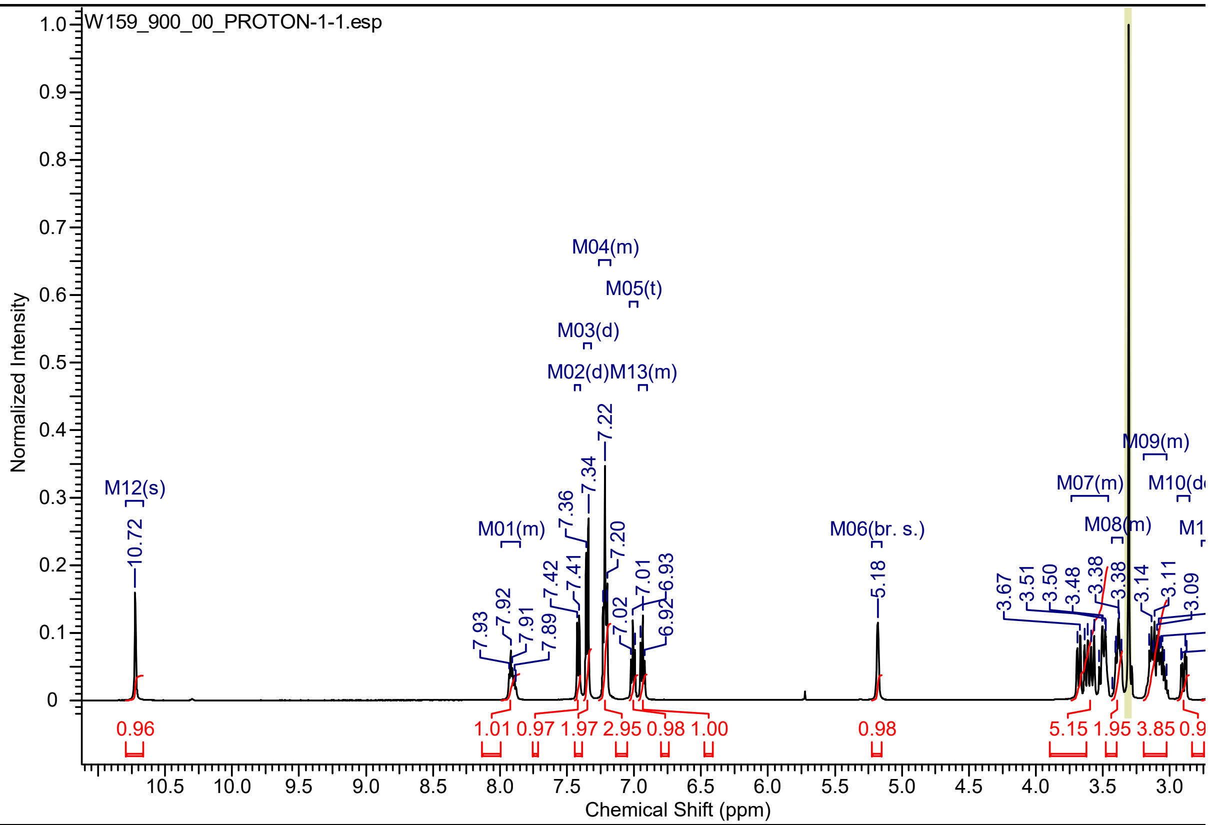 W159_900_00_PROTON-1-1.esp<br>Normalized Intensity<br>Chemical Shift (ppm)
**12**	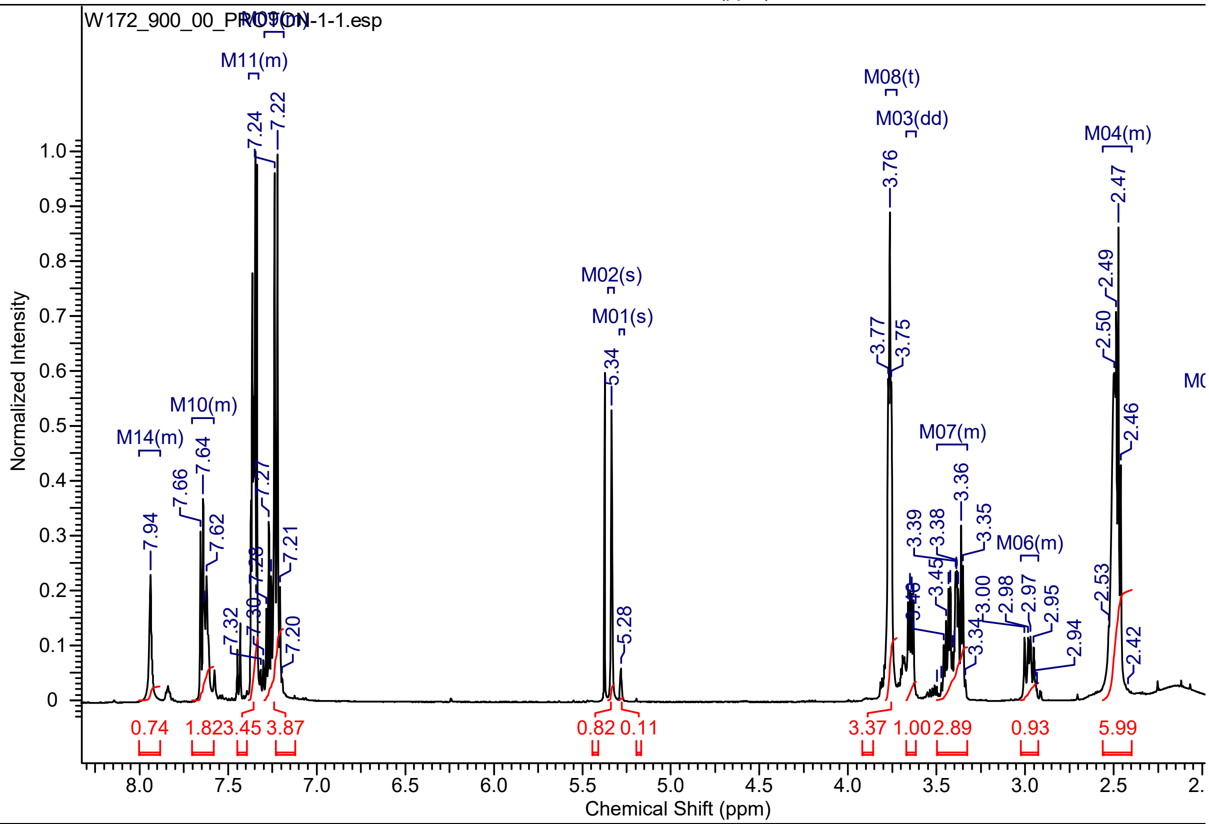 W172_900_00_PROTON-1-1.esp<br>Normalized Intensity<br>Chemical Shift (ppm)

**1*RS*,3*RS*-13**	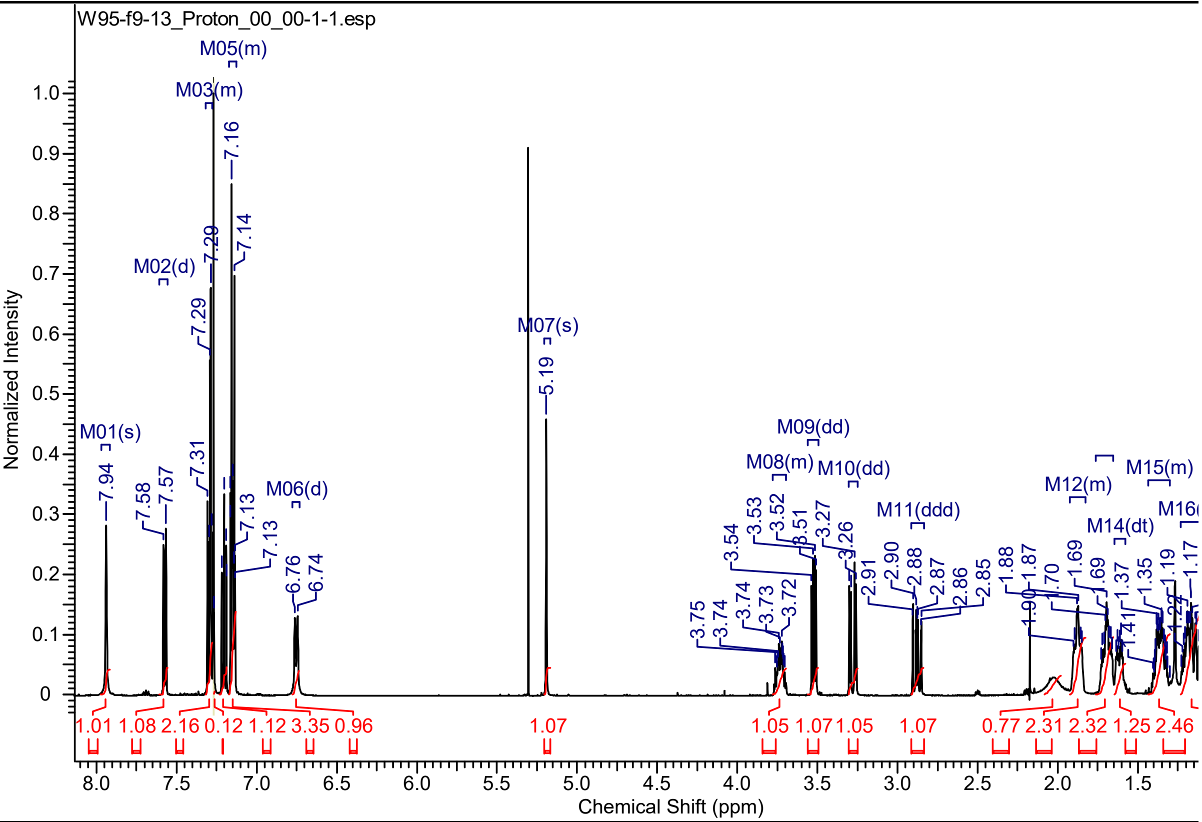
**1*RS*,3*SR*-13**	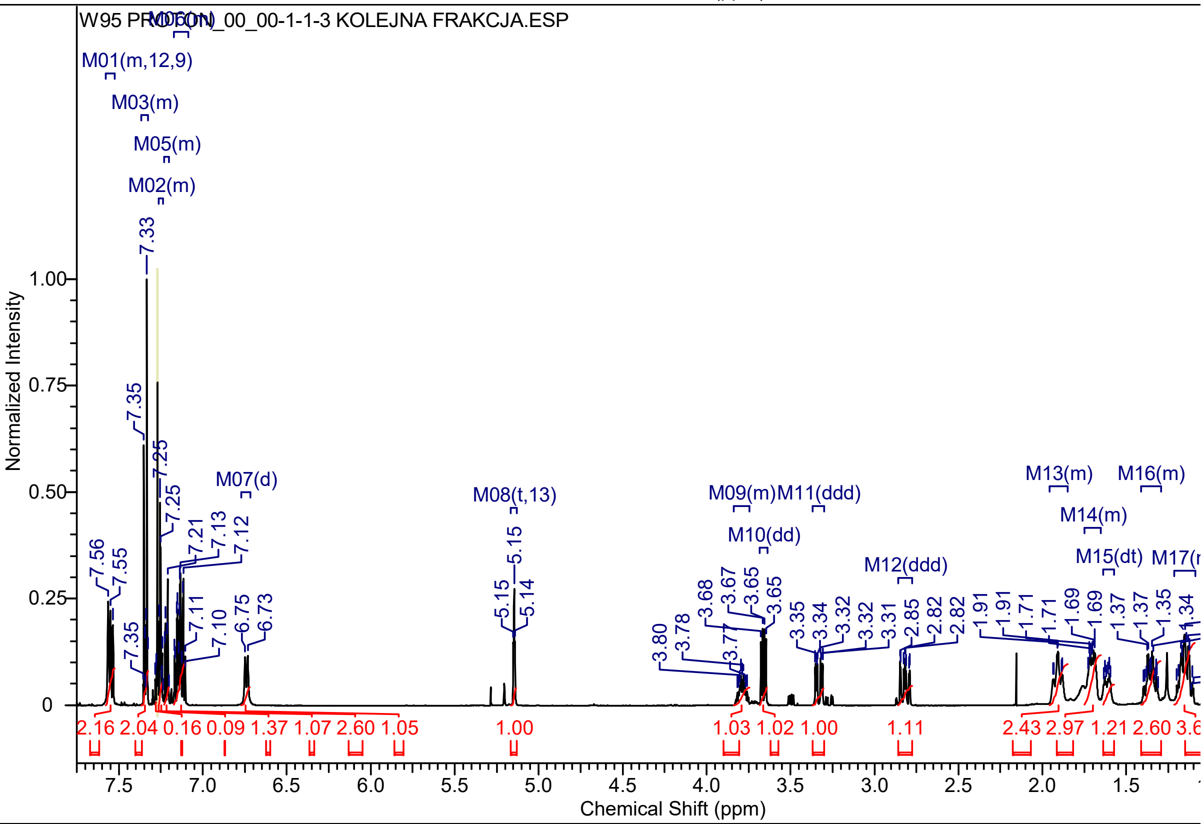

14

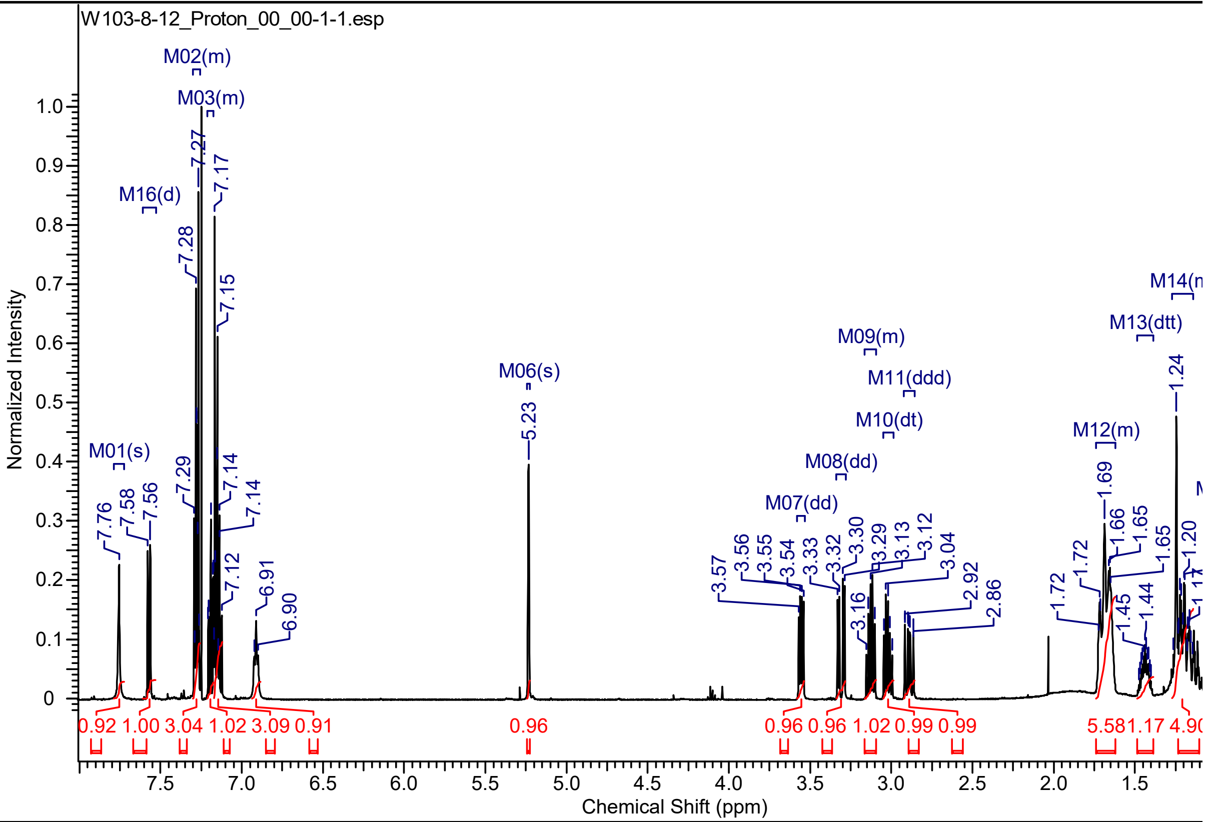
W103-8-12_Proton_00_00-1-1.esp
M01(s)
M02(m)
M03(m)
M06(s)
M07(dd)
M08(dd)
M09(m)
M10(dt)
M11(ddd)
M12(m)
M13(dtt)
M16(d)
7.76
7.58
7.56
7.29
7.28
7.27
7.17
7.15
7.14
7.14
7.12
6.91
6.90
5.23
3.57
3.56
3.55
3.54
3.33
3.32
3.30
3.29
3.16
3.13
3.12
3.04
2.92
2.86
1.72
1.72
1.69
1.66
1.65
1.65
1.45
1.44
1.24
1.20
0.92 1.00 3.04 1.02 3.09 0.91
0.96
0.96 0.96 1.02 0.99 0.99
5.58 1.17
Normalized Intensity
Chemical Shift (ppm)

^{13}C-NMR

2

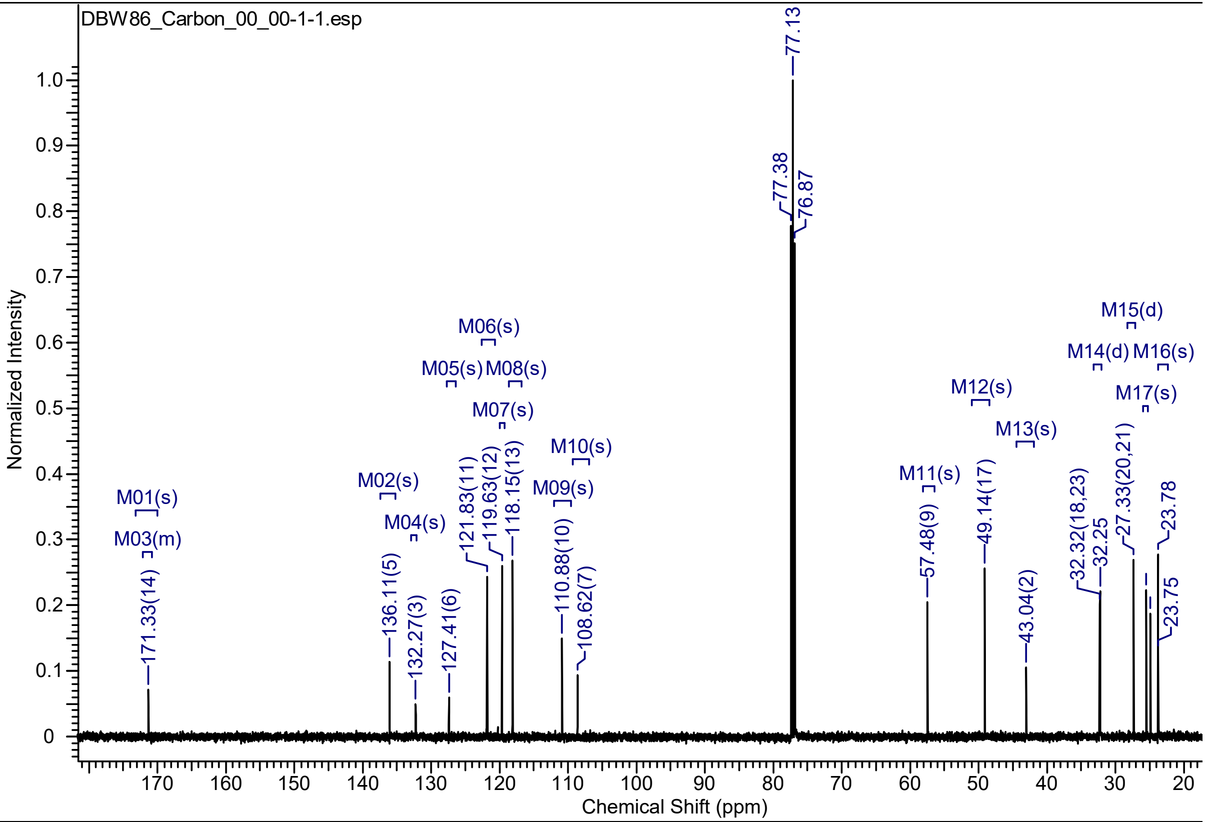
DBW86_Carbon_00_00-1-1.esp
M01(s)
M03(m)
M02(s)
M04(s)
M05(s)
M06(s)
M07(s)
M08(s)
M09(s)
M10(s)
M11(s)
M12(s)
M13(s)
M14(d)
M15(d)
M16(s)
M17(s)
171.33(14)
136.11(5)
132.27(3)
127.41(6)
121.83(11)
119.63(12)
118.15(13)
110.88(10)
108.62(7)
77.38
77.13
76.87
57.48(9)
49.14(17)
43.04(2)
32.32(18,23)
32.25
27.33(20,21)
23.78
23.75
Normalized Intensity
Chemical Shift (ppm)

7

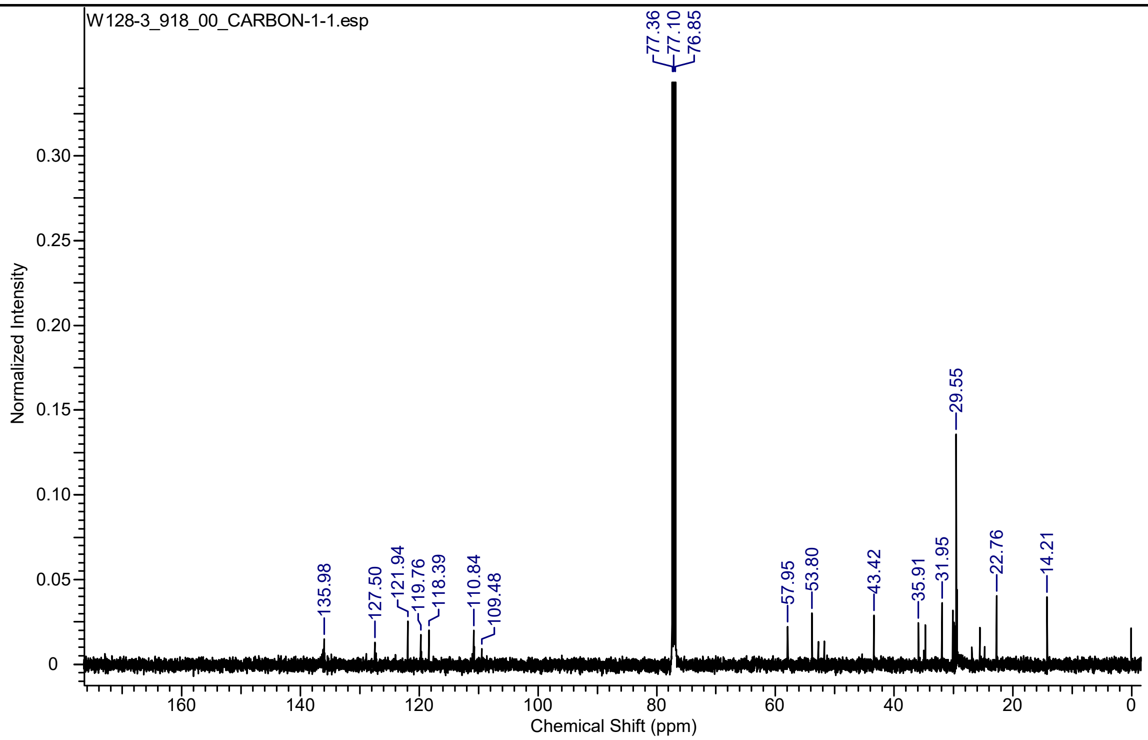

Due to fact that compound 7 was isolated as a mixture of racemic diastereomers on the ^{13}C NMR spectra we can observe extra signals from minor diastereomer.
